# Nanotechnology for microglial targeting and inhibition of neuroinflammation underlying Alzheimer’s pathology

**DOI:** 10.1186/s40035-023-00393-7

**Published:** 2024-01-04

**Authors:** Hoda M. Gebril, Aravind Aryasomayajula, Mariana Reis Nogueira de Lima, Kathryn E. Uhrich, Prabhas V. Moghe

**Affiliations:** 1 Department of Biomedical Engineering, Rutgers University, 599 Taylor Rd., Piscataway, NJ 08854 USA; 2 Department of Chemical and Biochemical Engineering, Rutgers University, 98 Brett Rd., Piscataway, NJ 08854 USA; 3 Department of Chemistry, University of California, 501 Big Springs Rd., Riverside, CA 92507 USA

**Keywords:** Amphiphilic nanoparticle, Microglia, Fibril amyloid beta, Alzheimer’s disease, Neuroinflammation, Scavenger receptor

## Abstract

**Background:**

Alzheimer's disease (AD) is considered to have a multifactorial etiology. The hallmark of AD is progressive neurodegeneration, which is characterized by the deepening loss of memory and a high mortality rate in the elderly. The neurodegeneration in AD is believed to be exacerbated following the intercoupled cascades of extracellular amyloid beta (Aβ) plaques, uncontrolled microglial activation, and neuroinflammation. Current therapies for AD are mostly designed to target the symptoms, with limited ability to address the mechanistic triggers for the disease. In this study, we report a novel nanotechnology based on microglial scavenger receptor (SR)-targeting amphiphilic nanoparticles (NPs) for the convergent alleviation of fibril Aβ (fAβ) burden, microglial modulation, and neuroprotection.

**Methods:**

We designed a nanotechnology approach to regulate the SR-mediated intracellular fAβ trafficking within microglia. We synthesized SR-targeting sugar-based amphiphilic macromolecules (AM) and used them as a bioactive shell to fabricate serum-stable AM–NPs via flash nanoprecipitation. Using electron microscopy, in vitro approaches, ELISA, and confocal microscopy, we investigated the effect of AM–NPs on Aβ fibrilization, fAβ-mediated microglial inflammation, and neurotoxicity in BV2 microglia and SH-SY5Y neuroblastoma cell lines.

**Results:**

AM–NPs interrupted Aβ fibrilization, attenuated fAβ microglial internalization via targeting the fAβ-specific SRs, arrested the fAβ-mediated microglial activation and pro-inflammatory response, and accelerated lysosomal degradation of intracellular fAβ. Moreover, AM–NPs counteracted the microglial-mediated neurotoxicity after exposure to fAβ.

**Conclusions:**

The AM–NP nanotechnology presents a multifactorial strategy to target pathological Aβ aggregation and arrest the fAβ-mediated pathological progression in microglia and neurons.

**Graphical Abstract:**

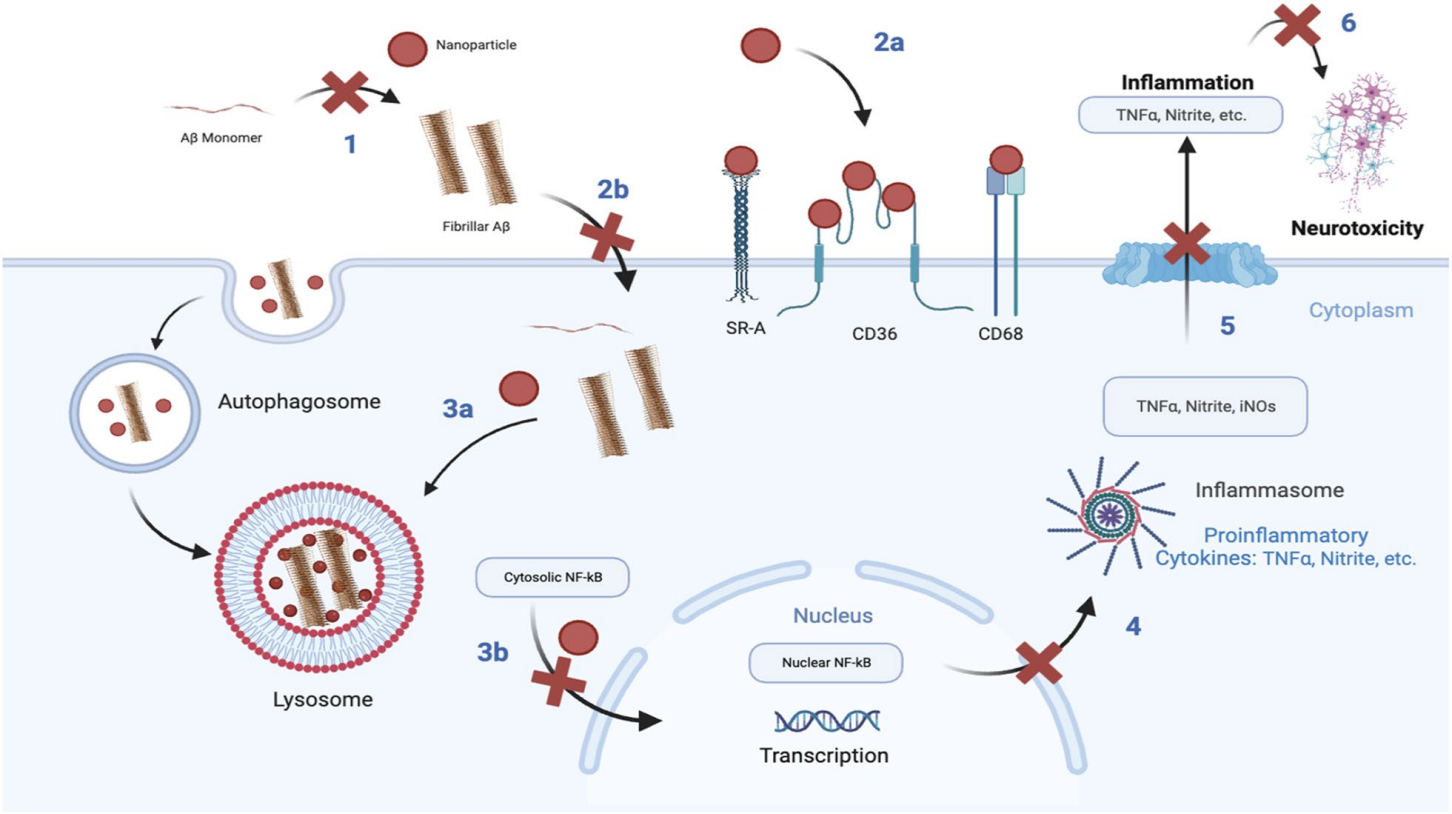

**Supplementary Information:**

The online version contains supplementary material available at 10.1186/s40035-023-00393-7.

## Background

Alzheimer’s disease (AD) is the sixth leading cause of death in the United States, which has an estimated population of 5.8 million individuals aged 65 or older living with AD [1]. As AD accounts for ~ 60% to 80% of the dementia cases, the socio-economic burden of the disease has grown by 35% since 2010 [1, 2]. The global cost of the disease is estimated to reach two trillion dollars by 2030 [2]. Early clinical stages of AD are associated with profound neuroinflammation, intracellular neurofibrillary tangles, and extracellular deposits of amyloid plaques of fibril β-amyloid (fAβ) [3–7]. fAβ is formed by aggregation of Aβ peptides that are generated via the proteolytic cleavage of amyloid precursor protein (APP) by β- and γ-secretases. While approximately 5% of AD cases are characterized by early onset of symptoms due to inheritable genetic mutations, the vast majority of AD cases are multi-factorial in etiology and characterized by sporadic late onset. Nevertheless, the single risk factor for sporadic AD is age [8]. This presents an immense health challenge, especially with the increasing longevity of the world’s growing population.

In AD patients, Aβ exists in the soluble oligomeric form [9] and as insoluble fibrils [10] in the parenchymal extracellular space. fAβ drives several cytopathological changes, including activation of glial cells such as microglia and astrocytes, which ultimately lead to irreversible neuronal damage [11, 12]. Currently, the FDA-approved non-curative AD drugs are used only to either delay the clinical decline in AD individuals or temporarily mitigate symptoms due to mild to moderate AD [13]. Under the recent accelerated approval pathway, the only FDA-approved curative drug was designed to remove fAβ deposits in early stages of AD [14]. Although this supports the hypothesis that fAβ is an early event in AD, the disease pathogenesis and etiology remain diverse and multifactorial due to the presence of other events such as aberrant tau accumulation and neuroinflammation [15, 16].

Microglia are the primary immune cells of the central nervous system (CNS), accounting for approximately 5%–15% of cells in the CNS [17]. They play a pivotal role in brain homeostasis, including neuronal maintenance, immune surveillance, and clearance of misfolded proteins, pathogens and cell debris [18]. In AD, the resident resting microglia are activated and migrate to regions of dense fAβ plaques to promote fAβ clearance. However, chronic activation of microglia leads to the release of pro-inflammatory cytokines and chemokines, which exacerbate the progression of the disease [19, 20]. A growing body of evidence suggests that functional and healthy microglia can efficiently restrict fAβ pathological overgrowth [21, 22]. In line with this notion, activated microglia have been linked to Aβ seeding and plaque growth [23–25]. Moreover, recent studies suggest that aging is associated with dysfunctional microglia [26–28]. Therefore, microglia can serve as a potential therapeutic target for age-related disorders including AD.

The physical association of microglia with fAβ in AD suggests the involvement of surface receptors in this interaction. Scavenger receptors (SRs) are structurally conserved membrane receptors highly expressed on macrophages, microglia, and endothelial cells. To date, several SRs have been associated with the pathogenesis of fAβ. Class A (SRA1) and B (CD36) SRs have been associated with the internalization of fAβ and the subsequent inflammatory response [29, 30]. Other receptors associated with fAβ pathogenesis are CD14, TLR2, and TLR4 [31]. Inhibition of the CD36–fAβ interaction has been found to halt the fAβ-mediated microglial immune response [32]. Moreover, in vivo studies in CD36-deficient mice reported reduction of the fAβ-mediated oxidative stress [33] and microglial recruitment [34]. Importantly, SRA1 deficiency [35] as well as its complete knockout [36] resulted in a significant reduction of fAβ microglial uptake. Other SRs such as Class D SR, CD68, which is expressed on lysosomes and endosomes of microglia, are overexpressed in human AD [37]; however, their role in fAβ trafficking has not been systematically elucidated. The reported effects of SRs on the fAβ-mediated pathology make them potential candidates for therapeutic applications.

Research on synthetic compounds that possess the physical properties of SR-binding ligands has been reported [38–40]. Approaches targeting CD36 have been explored as a means to ameliorate neuroinflammation associated with neurodegenerative disorders. While small-molecule ligands to block the interactions between CD36 and fAβ have shown promising results [41, 42], their low solubility in water, short half-life, and low bioavailability have limited their application [43]. Advances in nanomedicine have led to generation of new classes of therapeutics against CNS disorders, with high biocompatibility, bioavailability, and structural stability to ensure targeted delivery [44]. Nanoparticles and nanocarrier-based approaches have been used to target the fAβ-mediated pathology [45–47]. However, most of these approaches were designed to target one aspect of the disease pathology.

We have previously designed a new class of amphiphilic macromolecules (AMs) composed of sugar-based backbones, aliphatic side chains, and hydrophilic poly (ethylene glycol) (PEG); these were conceived to be biomimetic synthetic ligands for lowering the SR–α-synuclein (α-syn) interactions and the subsequent microglial inflammatory response [48–50]. Due to their amphiphilic nature, AMs can be complexed around hydrophobic core molecules via kinetic flash nanoprecipitation (FNP), forming nanoparticles (NPs). The AM-based NPs demonstrated improved bioactivity, stability, and resistance to AM release in serum-rich environment [51, 52].

In this study, by focusing on the microglial SR-mediated internalization of fAβ as a therapeutic target in the pathway of fAβ-mediated microglial inflammation, we set out to test the effects of SR-binding AM-NPs on Aβ fibrilization, fAβ internalization, lysosomal degradation, and fAβ-mediated microglial inflammation. Our central hypothesis is that the AM-NPs could counteract the fAβ internalization and the subsequent pro-inflammatory response and neurotoxicity in a convergent manner. To test this hypothesis, we investigated the roles of two AM shell compositions from our library, tartaric acid-derived (T_12_P_5_) and mucic acid-derived (M_12_P_5_), in the fAβ-mediated pathology. We first elucidated their effect on the fibrilization of Aβ in a cell-free system, and second, investigated the effects of the AM-NPs on fAβ microglial internalization, degradation, inflammatory response, and neurotoxicity.

## Methods

### Synthesis of AMs

The macromolecular shells M_12_P_5_ and T_12_P_5_ were synthesized via esterification reactions conducted in two steps as previously described [48, 53, 54]. Briefly, the first step of M_12_P_5_ synthesis was performed by reacting mucic acid (20 mmol), zinc chloride (2 mmol) and lauroyl chloride (160 mmol) at 90 °C under inert atmosphere for 12 h. Diethyl ether (20 ml) was added to the reaction mixture after it cooled to room temperature and the mixture was poured over ice-cold water (150 ml) under stirring. Then 80 ml of diethyl ether was added to the mixture and stirred continuously for 30 min. Extractions with brine were performed until the aqueous layer reached pH ~ 7. The organic layer was separated, dried over sodium sulfate, and evaporated under reduced pressure. Purification was performed by dissolving the crude product in diethyl ether (20 ml) and precipitation into petroleum ether (200 ml). The pure product (M_12_) was isolated by vacuum filtration. For the first step of T_12_P_5_ synthesis, tartaric acid (7 mmol) and zinc chloride (2.2 mmol) were suspended in lauroyl chloride (52.5 mmol) and allowed to stir for 24 h under inert atmosphere at 95 °C. Then, DI-water (30 ml) and diethyl ether (100 ml) were added to quench the reaction and stirring was continued for 30 min at room temperature. Five extractions using DI-water (100 ml/wash) were performed. The organic layer was separated, dried over magnesium sulfate, and concentrated under reduced pressure. The brown liquid obtained was precipitated over hexanes and pure product (T_12_) was isolated by vacuum filtration. The second step of the synthesis consisted of the PEGylation reaction of M_12_ or T_12_ via carbodiimide chemistry to generate M_12_P_5_ or T_12_P_5_, respectively. For the PEGylation reaction, M_12_ or T_12_ (0.45 mmol) and DPTS (0.15 mmol) were dissolved in anhydrous dichloromethane (DCM, 10 ml) and anhydrous dimethylformamide (DMF, 3 ml) under inert atmosphere at room temperature. mPEG (5 k) was added to the reaction mixture and after complete dissolution, *N*,*N*-diisopropylcarbodiimide (DIC, 0.48 mmol) was added in a drop-wise manner and stirring was continued for 48 h under argon. Next, the reaction mixture was cooled down at − 20 °C and the white solid (side product) was precipitated and removed by vacuum filtration. Extra DCM (25 ml) was added to the filtrate and extractions with hydrochloric acid HCl (0.1 M, 1 × 40 ml) and brine (2 × 40 ml) were performed as part of the purification. After separation from the aqueous layer, the organic layer was dried over magnesium sulfate and solvent was evaporated under reduced pressure. The product was dissolved in diethyl ether (50 ml) and isolated by centrifugation (1370*g*, 5 min). Products were dried under vacuum and characterized using ^1^H NMR-spectroscopy, FTIR spectroscopy and differential scanning calorimetry.

### Nanoparticle fabrication and characterization

**Fig. 1:**
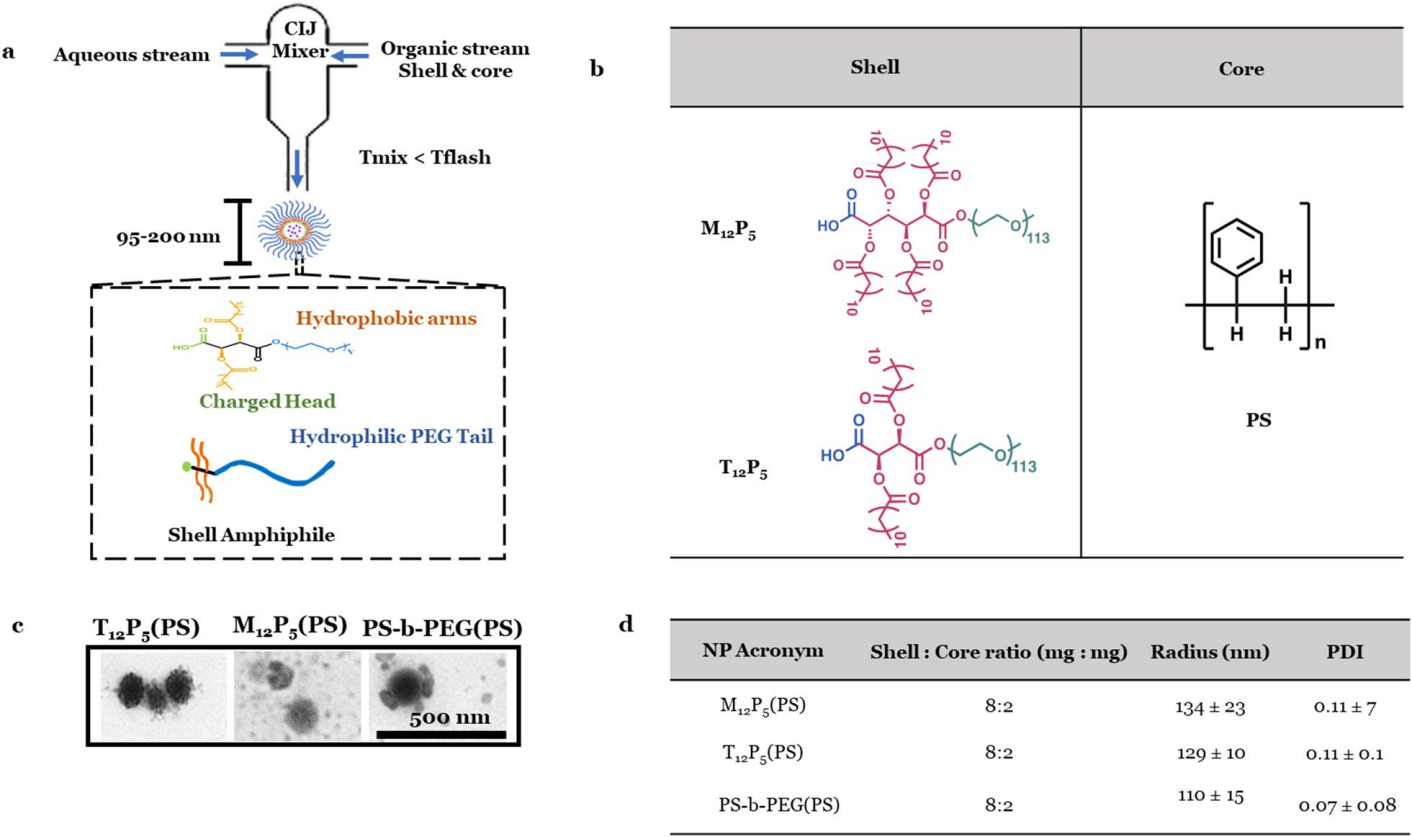
Nanoparticle fabrication via flash nanoprecipitation (FNP). **a** Schematic diagram of the FNP procedure for the synthesis of AM-nanoparticles (NPs). A confined impinging jet mixer (CIJ) was used to mix a stream of 250 µl of 50% (*v*/*v*) mixture of tetrahydrofuran (THF) containing 8 mg/ml shell molecule and 2.5 mg/ml hydrophobic core molecule with 250 µl of an aqueous stream. Key element of FNP technique for the fabrication of stable NPs is the induction time of aggregation and precipitation. The time required for a complete and homogenous mixing of the aqueous and solvent stream is Tmix, while the precipitation time of NPs is Tflash. **b** Table listing the chemical structures of AM shell molecules and polystyrene core. **c** Transmission electron microscope (TEM) images of NPs. **d** Table summarizing the characterization of NP radius and polydispersity index (PDI). Data are presented as mean ± SEM; *n* = 3

NPs were synthesized and characterized as previously detailed using established techniques [48, 53]. NPs were fabricated using the FNP technique (Fig. 1a) as previously reported [48, 51, 52]. In brief, a confined impinging jet mixer was used to mix a stream of 250 μl of 50% (*v*/*v*) mixture of tetrahydrofuran (Sigma, St. Louis, MO) containing 8 mg/ml shell molecule and 2.5 mg/ml hydrophobic core molecule with 250 μl of an aqueous stream. To ensure homogenous NP size distribution, the time of NP formation (Tflash) was prescribed to be more than the time of mixing both streams (Tmix). For fluorescent NPs, 3.75% of 1,1′-dioctadecyl-3,3,3,3-tetramethylindocarbocyanine perchlorate (Dil) (Thermo Fisher, Waltham, MA) was mixed with the organic stream of shell and core mixture. The exit stream was immediately introduced into a ninefold volume of water, then NPs were dialyzed against water using a 3.5-kDa MW cutoff dialysis cassette (Thermo Fisher). The hydrodynamic radius and polydispersity index of NPs were characterized using dynamic light scattering (DLS) (a Malvern-Zetasizer Nano ZS90 series DLS detector) [51]. The critical micelle concentration, size, and charge data have been published in the literature [48, 51, 55].

### Preparation and characterization of Aβ_1-42_ fibrils

Aβ_1-42_ fibrils were generated as previously reported with slight modifications [56, 57]. Briefly, Aβ_1–42_ (Anaspec, Fremont, CA) was suspended in 100% 1,1,1,3,3,3-hexafluoro-2-propanol to a final concentration of 5 mg/ml, aliquoted and then dried at room temperature overnight in a fume hood. The aliquoted peptide was dissolved in DMSO to a final concentration of 5 mM and sonicated for 1 min in low-binding microcentrifuge tubes (Corning, Manassass, VA). The Aβ_1−42_ peptide was diluted in phosphate-buffered saline (PBS) and 0.2% sodium dodecyl sulfate to 200 µM; next, fibrils were prepared by incubating the diluted peptide for at least 4 weeks at 37 °C with constant shaking at 300 rpm. Fibrils were separated after centrifugation for 1 h at 5000*g* at 4 °C. The concentration of Aβ fibrils was determined by measuring absorbance at 280 nm and calculated using extinction coefficient at 1280 M^−1^ cm^−1^ [58]. Fibril formation was verified using thioflavin-T (Th-T) fluorescence assay and transmission electron microscopy (TEM).

### Th-T fluorescence assay

Aβ fibril formation was assessed by the Th-T fluorescence assay [59]. First, 3 mM of Th-T (Sigma) stock was prepared and filtered through a 0.2-µm syringe filter. To measure Aβ fibril formation, 40 µM of Th-T was mixed with 5 µl of either PBS (control) or the protein. Using a black-bottom 96-well microplate (Corning), the fluorescence intensity was measured at room temperature using a Tecan Infinite M200 Pro microplate reader at excitation 450 nm and emission 485 nm. Results are presented as a ratio to the fluorescence intensity of the control samples of PBS in Th-T.

### TEM

To prepare fAβ samples for TEM imaging, a negative staining protocol was conducted as described before. Briefly, 5 μl of 20 μM fAβ was loaded on a formvar-coated, carbon-stabilized copper grid (400 mesh, Pacific Grid-tech, San Francisco, CA). Excess solution was drained using a Whatman filter paper. The grid was washed and negatively stained with 5 μl of 2% uranyl acetate. Excess solution was drained, and the grid imaged using a Philips CM12 electron microscope with an AMT-XR11 digital camera. The images were acquired at magnifications of 60,000 at 80 kV. For NP imaging, NPs were loaded on a formvar-coated, carbon-stabilized copper grid (400 mesh, Pacific Grid-tech). Excess solution was drained using a Whatman filter paper, and then NPs were directly imaged. To image the preformed fAβ in the presence of AM-NPs, a mixture of fAβ and AM-NPs was loaded on a formvar-coated, carbon-stabilized copper grid, washed, and negatively stained as described above. The images were acquired at magnifications of 22,000 at 80 kV.

### Kinetics of Aβ fibrillization

The effect of AM-NPs on Aβ fibrilization was assessed as previously described [59]. Samples containing 8 µM of monomeric Aβ in the absence or presence of NPs (1:10, *v*/*v*) were loaded with 20 µM Th-T into 96-well clear bottomed non-binding half-area plates (Corning). NPs only mixed with Th-T were included as a negative control to measure any background reading of NPs. Plates were sealed with the Axygen sealing tape (Corning) and the fluorescence intensity was monitored over 63 h using a Tecan Infinite M200 Pro microplate reader with excitation at 450 nm and emission at 485 nm while agitated at 600 rpm at 37 °C. After subtraction of NP background fluorescence, each sample containing Aβ and NPs was normalized to the Aβ fluorescence.

### Cell culture

#### BV2 mouse microglia cell line

BV2 microglia were kindly provided by Drs. Bin Liu (University of Florida) and Jason Richardson (Northeast Ohio Medical University). The BV2 microglia were cultured in Dulbecco’s Modified Eagle Medium (DMEM) (Gibco, Manassass, VA) supplemented with 10% fetal bovine serum (FBS) and 1% penicillin–streptomycin (Pen/Strep) (Gibco). Cells were plated in a 96-well plate at 20,000/well and allowed to adhere to plates for 24 h before any treatment. All treatments were conducted in DMEM medium containing 1% FBS and 1% Pen/Strep.

#### SH-SY5Y human neuroblastoma cell line

SH-SY5Y (ATCC, Manassas, VA) cells were plated in a 96-well plate at 15,000 cells/per well and allowed to adhere overnight in DMEM supplemented with 10% FBS and 1% Pen/Strep. All treatments were conducted in DMEM medium containing 1% FBS and 1% Pen/Strep.

### Cell-based competitive receptor binding assay

To screen for SRs that competitively bind to our AM-NPs, we tested the efficacy of AM-NPs to compete with CD36-, CD68-, SRA1-, and TLR2-specific antibodies on the BV2 cell surface. The BV2 microglia were plated at 20,000 cells/well in a 96-well plate and cultured for 24 h. Using DMEM containing 0.2% sodium azide, the cells were incubated with NPs for 1 h, then co-incubated with SR-specific antibody or isotype control for 30 min. The cells were fixed with 4% paraformaldehyde (PFA) (Sigma) for 15 min, washed twice with PBS, and then blocked with 2% goat serum without Triton-X-100 to avoid plasma membrane permeabilization. To avoid internalization of NPs or antibodies, cells were incubated on ice and in the presence of sodium azide. Cells were then incubated with Alexa 488- or 594-conjugated secondary antibody (Life Technologies, Carlsbad, CA) for 1 h at room temperature. The cells were washed with PBS and then counter-stained with Hoechst (Thermo Fischer) to visualize nuclei. Cells untreated with NPs were used as a control for each SR. The primary antibodies were CD36 (Abcam, Fremont, CA), CD68 (Biolegend, San Diego, CA), SRA1 (Proteintech, Rosemont, IL), and TLR2 (Novus, St. Charles, MO). Cells were imaged on a Zeiss LSM 780 confocal microscope using a 20 × objective. Extracellular fluorescence quantification was performed using the FIJI/ImageJ software by applying the thresholding technique that determines the foreground pixels over that background pixels at a fixed threshold value. The fluorescence of each field was divided by the number of cells in the field and then normalized to the control.

### Aβ internalization assay

To screen for SRs that mediate fAβ internalization and to test the hypothesis that AM-NPs modulate fAβ internalization in BV2 cells, microglia were plated at 20,000 cells/well in a 96-well plate. The BV2 microglia were co-incubated with 20 μM of fAβ mixed with 0.035 wt% HiLyte Fluor 488-labeled fAβ (for imaging purposes) for 24 h after pre-incubation in the presence or absence of SR-specific antibodies, isotype controls, or AM-NPs for 24 h. Cells were fixed with 4% PFA and then washed twice with PBS to remove extracellular fAβ. Cells were then incubated with 0.5% Triton-X-100 (Sigma) in potassium buffered saline (PBS-T) to remove any membrane-bound fAβ particles. The primary antibodies used were anti-CD36 (Abcam), anti-CD68 (Biolegend), anti-SRA1 (Proteintech), and anti-TLR2 (Novus) antibodies. The cells were imaged with a Zeiss LSM 780 confocal microscope using a 20 × objective. Intracellular fluorescence quantification was performed using the FIJI/ImageJ software by applying the thresholding technique that determines the foreground pixels over that background pixels at a fixed threshold value. For untreated cell controls, fluorescence images were segmented based on cell boundaries visualized in bright-field images.

### Thioflavin-S assay

BV2 microglia were co-incubated with 20 μM fAβ for 24 h after pre-incubation in the presence or absence of AM-NPs for 24 h. Cells were fixed with 4% PFA and then washed two times with PBS to remove extracellular fAβ. Cells were then incubated with 0.5% Triton-X-100 (Sigma) in potassium buffered saline (PBS-T) to remove any membrane-bound fAβ particles. Fixed cells were incubated with 0.01% Thioflavin-S stain (Sigma) for 30 min, then washed one time with 50% ethanol. Cells were imaged on a Zeiss LSM 780 confocal microscope using a 20× objective. Intracellular fluorescence quantification was performed using FIJI/ImageJ software by measuring the mean grey value in cells segmented by applying the same fluorescence thresholds to all collected images.

### Tumor necrosis factor-alpha (TNF-α) and nitric oxide (NO) assay

BV2 microglia were plated at 20,000 cells/well in a 96-well plate and allowed to adhere overnight. The cells were co-treated with 20 µM fAβ in the presence or absence of NPs or 10 ng/ml lipopolysaccharide (LPS). After 24 h, the supernatant was collected and assayed for TNF-α production using ELISA (R&D systems, Minneapolis, MN). NO production in the supernatant was measured using Griess reagent (Promega, Madison, WI). The cells were fixed in 4% PFA, washed with PBS, and then intracellular inducible nitric oxide synthase (iNOS) was assayed in immunocytochemistry as described below.

### Immunocytochemistry

Cells were fixed with 4% PFA for 15 min, then washed with PBS. Cells were permeabilized in PBS-T for 10 min, then blocked for 1 h with 2% goat serum (MP Biomedicals, Irvine, CA) blocking buffer at room temperature. The cells were incubated with primary antibodies including anti-iNOS (Abcam), and anti-nuclear factor-κB (NF-κB) (Santa Cruz, Santa Cruz, CA) overnight at 4 °C, washed with PBS-T, and then incubated with Alexa 488- or 594-conjugated secondary antibody (Life Technologies) for 1 h at room temperature. The cells were washed with PBS and then counterstained with Hoechst (Thermo Fischer) to visualize nuclei. For imaging extracellular or cell surface proteins, Triton-X-100 was eliminated from the protocol. Cells were imaged with a Zeiss LSM 780 confocal microscope using a 20 × or a 40 × water immersion objective.

### Lysosome activity

To study the activity of the acidic lysosomes, BV2 microglia were pre-treated with or without NPs for 24 h, then co-treated with fAβ488 for either 2 h or 24 h. The cells were then washed and incubated in 70 µM LysoTracker red DND-99 (ThermoFisher) for 30 min prior to fixation with 4% PFA. Images were captured at multiple focal planes via a Zeiss LSM 780 confocal microscope using a 20 × or a 40 × objective. The colocalization of fAβ488 with lysosomes was analyzed using the Mander’s overlap coefficient as previously described [60]. For CD68 (Biolegend) and the lysosomal associated membrane protein (LAMP)-1 (Invitrogen) immunostaining, BV2 microglia were pre-treated with or without NPs for 24 h, followed by co-treatment with 20 µM fAB488 and Dil-labeled NPs for 2 h. Immunostaining was performed as described above.

### Autophagic activity

To study the autophagic activity, immunostaining and electron microscopy were conducted [61, 62]. BV2 microglia were pre-treated with or without NPs for 24 h, and then co-treated with fAβ for 30 min prior to fixation with 4% PFA for immunocytochemistry. Cells were stained against the autophagosome marker LC3B (Invitrogen) and nuclei were counter-stained using Hoechst according to the immunocytochemistry protocol described above. Images for immunocytochemistry were captured at multiple focal planes via a Zeiss LSM 780 confocal microscope using a 40 × objective. LC3 intensity was determined in the cytoplasm by first defining the cell nucleus using Hoechst stain channel, and then subtracting the nuclei fluorescence from the whole cell fluorescence.

For electron microscopy, cells were fixed with a mixture of 2.5% glutaraldehyde and 4% PFA in 0.1 M cacodylate buffer at pH 7.4 and then post-fixed in buffered 1% osmium tetroxide. Cell pellets were dehydrated in graded acetone series and then embedded in EMbed resin (Electron Microscopy Sciences, Hatfield, PA). Using a diamond knife on a Leica Ultracut EM Ultramicrotome (Leica Microsystems, Deerfield, IL), ultrathin (90 nm) sections were collected on coated 200-mesh grids and stained with saturated solution of uranyl acetate and lead citrate. Grids were imaged using an AMT XR111 digital camera (Advanced Microscopy Techniques, Woburn, MA) at 80 kV on a Philips CM12 electron microscope.

### NF-κB translocation assay

To investigate the nuclear translocation of NF-κB in BV2 cells, an immunostaining assay was conducted as previously described using confocal microscopy [63]. Briefly, BV2 cells were treated with 20 µM fAβ in the presence or absence of AM-NPs for 2 h and then immediately fixed with 4% PFA. Using the immunocytochemistry assay described above, the focal location of the cell nucleus was determined with Hoechst staining and used to define the nuclear region of interest (ROI). The mean nuclear NF-κB fluorescence intensity was measured in ROI while the cytoplasmic NF-κB fluorescence intensity was measured by subtracting ROI from the imaging field. Then the nuclear to cytoplasmic NF-κB fluorescence ratio was calculated.

### Image acquisition

Images of BV2 cells were obtained with a Carl Zeiss LSM 780 confocal microscope. Lasers for image acquisition were Argon Ion for Alexa Fluor 488 nm probe and HeNe for Alexa Fluor 647 nm probe configuration. Complementary brightfield was used for focusing, imaging, and analysis purposes. For each configuration, detectors were adjusted to eliminate spectral overlap between channels to unique bandwidth. To further minimize the spectral bleed-through, images were taken for each fluorescent probe in sequential mode.

### Neurotoxicity assay

BV2 microglia were plated in a 96-well plate at 20,000 cells/well, and SH-SY5Y cells were plated separately in a 96-well plate at 15,000 cells/well. Microglia were treated with 20 µM of fAβ in the presence or absence of NPs for 24 h. In parallel, other wells were either treated with vehicle (1% FBS media plus PBS) or with 10 ng/ml LPS. The BV2-conditioned media (CM) from this experiment were harvested and used to treat SH-SY5Y cells for 24 h. The neurotoxicity in response to BV2-CM was quantified in SH-SY5Y cells using the lactate dehydrogenase (LDH) assay (Promega) and normalized to the fAβ-treated SH-SY5Y cells and to the SH-SY5Y cells exposed to CM of untreated BV2 cells.

### Statistical analysis

Data are presented as mean ± SEM unless otherwise indicated, from at least 3 independent experiments (*n* ≥ 3). Analysis was performed using student’s *t* test, one-way analysis of variance (one-way ANOVA), or two-way ANOVA followed by pairwise multiple comparisons test.* P* < 0.05 was considered as statistically significant.

## Results

### AM-NPs prevent Aβ fibrilization

Three sets of AM-NPs at equivalent concentrations with identical polystyrene (PS) core and comparable shell molecular mass concentrations were synthesized (Fig. 1a, b). The first set (T_12_P_5_(PS)) was composed of the bioactive tartaric acid-derived shell (T_12_P_5_) and the non-bioactive PS core. The second set (M_12_P_5_(PS)) was composed of the bioactive nucleic acid-derived shell (M_12_P_5_) and the non-bioactive PS core. The third set (PS-*b*-PEG(PS)) was composed of the equivalently sized control NPs composed of the non-bioactive polystyrene-*block*-poly (ethylene glycol) (PS-*b*-PEG) shell with a comparable molecular weight and the PS core (Fig. 1a, b). The NPs were fabricated via FNP (Fig. 1a), where the fast mixing speed allows the formation of nano-assemblies. Using different shell-to-core weight ratios, the optimal ratio that ensures the formation of non-aggregating and stable particles was used (Fig. 1c). Our results showed that at the shell-to-core ratio of 8:2, stable NPs with radii ranging from 90 to 200 nm were generated (Fig. 1c). Those NPs were characterized by a low polydispersity index, less than 0.3, indicating relatively monodisperse distribution in an aqueous solution (Fig. 1c).

**Fig. 2:**
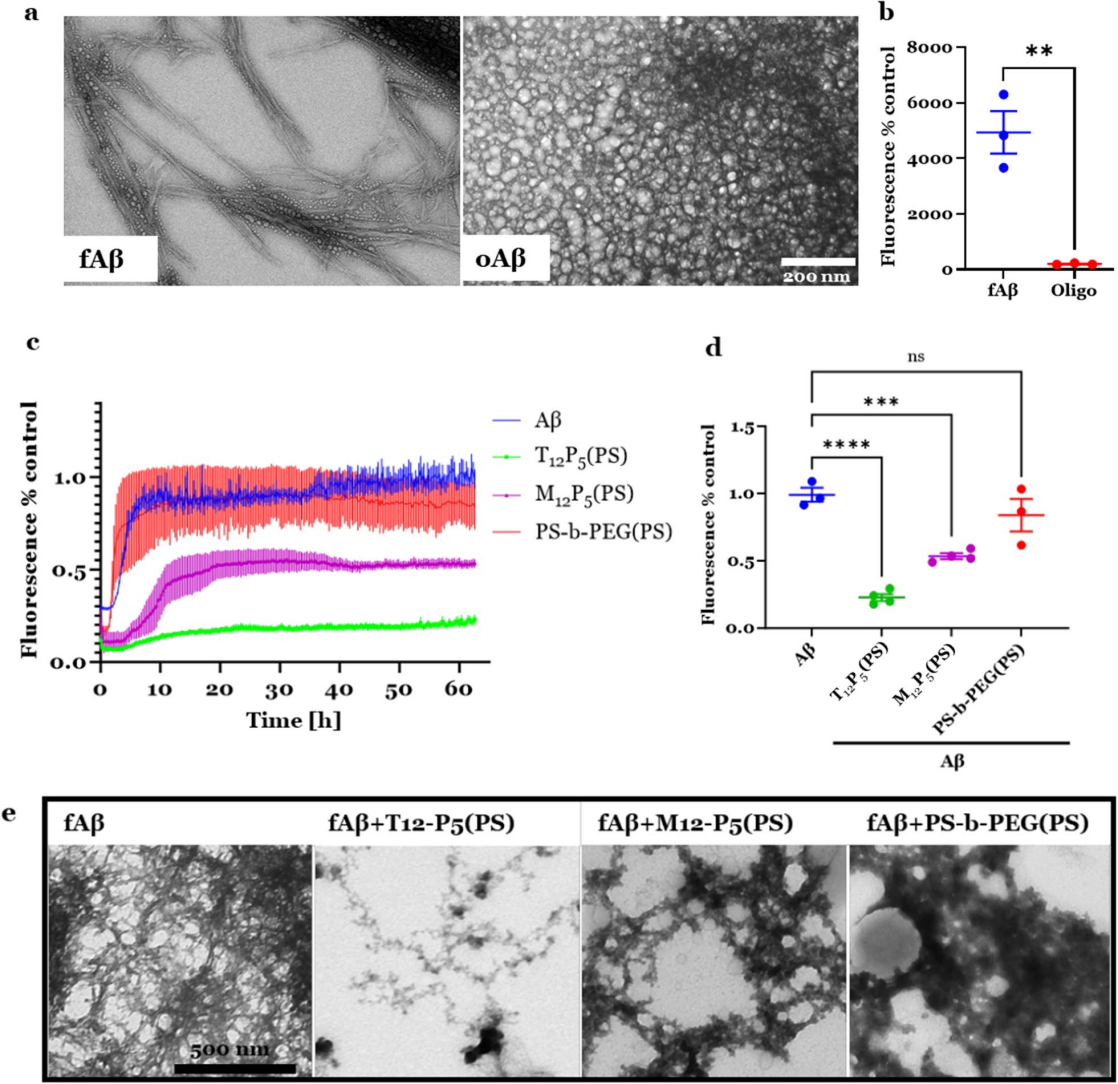
Development of fibril amyloid beta (fAβ) and the effect of NPs on Aβ fibrilization kinetics. **a** Representative transmission electron microscope (TEM) images of fAβ and oligomeric Aβ (oAβ). fAβ or oAβ (both 5 μl) was loaded on a formvar-coated, carbon-stabilized copper grid. Scale bar, 200 nm. **b** Thioflavin-T (Th-T) assay for fAβ and oAβ to validate the aggregation of β sheets. Protein or PBS (control) was mixed with 40 µM Th-T in a black-bottom 96-well microplate. Data are presented as mean ± SEM; *n* = 3; **P = 0.003 for student’s *t* test. **c** Th-T kinetic assay conducted with Aβ monomer over 65 h at 37 °C. Samples containing monomeric Aβ only or in the presence of NPs were loaded with 20 µM Th-T into a 96-well clear-bottomed non-binding half-area plate. **d** Endpoint Th-T fluorescence at 63 h. Data are presented as mean ± SEM; *n* = 3–4; ****P < 0.0001 for T_12_P_5_(PS) versus fAβ488 and ***P = 0.0007 for M_12_P_5_(PS) versus fAβ488 for Dunnett’s multiple comparisons shown on graph by one-way ANOVA. **e** TEM images of preformed fAβ mixed with AM-NPs or distilled water for 2 h. Scale bar, 500 nm

Next, we synthesized fAβ in vitro from human Aβ_1-42_ peptide, the most abundant form in brain plaques [64, 65], using an established protocol with some modifications [66]. The ultrastructure of the produced fAβ was verified and differentiated from the soluble oligomeric Aβ (oAβ) using TEM (Fig. 2a), and the degree of fibrilization of fAβ was assessed using the Th-T assay (Fig. 2b). Next, to test the hypothesis that AM-NPs can interrupt the fibrilization of Aβ peptide, soluble Aβ peptide was incubated with Th-T in the presence or absence of NPs, T_12_P_5_ (PS), M_12_P_5_(PS), or control PS-*b*-PEG(PS), at 37 °C. Aromatic compounds such as thioflavin T/S and Congo red can selectively bind to β-sheet-rich fibrils; therefore, they have been widely used to probe Aβ fibrils in AD brain tissues both in vivo and in vitro [67, 68]. The binding of Th-T to fibrillar β sheets of fAβ stops the bond rotation of Th-T, and therefore, recovers its emission at 485 nm once excited [69, 70]. Our results showed the fibrilization kinetics of Aβ in Th-T (Fig. 2c), which is characterized by the commonly observed sigmoidal growth curve of Aβ fibrilization, which composed of three phases: lag phase, growth phase, and an equilibrium phase [71]. Interestingly, AM-NPs with bioactive shells T_12_P_5_(PS) and M_12_P_5_(PS), significantly shortened the growth phase of Aβ fibrilization and slowed the aggregation kinetics (Fig. 2c). In contrast, the control NP PS-*b*-PSG(PS) did not impact the kinetics of Aβ aggregation (Fig. 2c). Also, T_12_P_5_(PS) and M_12_P_5_(PS) significantly reduced the endpoint fluorescence of Th-T at 65 h by 76% (*P* < 0.0001) and 45% (*P* = 0.0007), respectively, compared to the control fAβ samples in Th-T (Fig. 2d). These results suggest that the bioactive shells on AM-NPs are sufficient to slow the transition from Aβ monomers to mature fibrils. Moreover, as AM-NPs significantly reduced the endpoint fluorescence of Th-T, which is reported to be directly proportional to the fibril content [69], these data indicate that the bioactive shells are able to reduce the amount of fAβ formed.

To validate this result, we investigated the ultrastructure of the preformed fAβ in the presence or absence of AM-NPs. Preformed fAβ was mixed with AM-NPs or distilled water for 2 h and then imaged using electron microscopy (Fig. 2e). As expected, AM-NPs, especially T_12_P_5_(PS), showed remarkable disaggregating effects on fAβ conformation (Fig. 2e), while fAβ showed amorphous structures in the presence of control PS-*b*-PEG(PS). These data suggest a remodeling effect of T_12_P_5_(PS) on fAβ.

### AM-NPs bind to fAβ-binding SRs and interrupt fAβ internalization

**Fig. 3:**
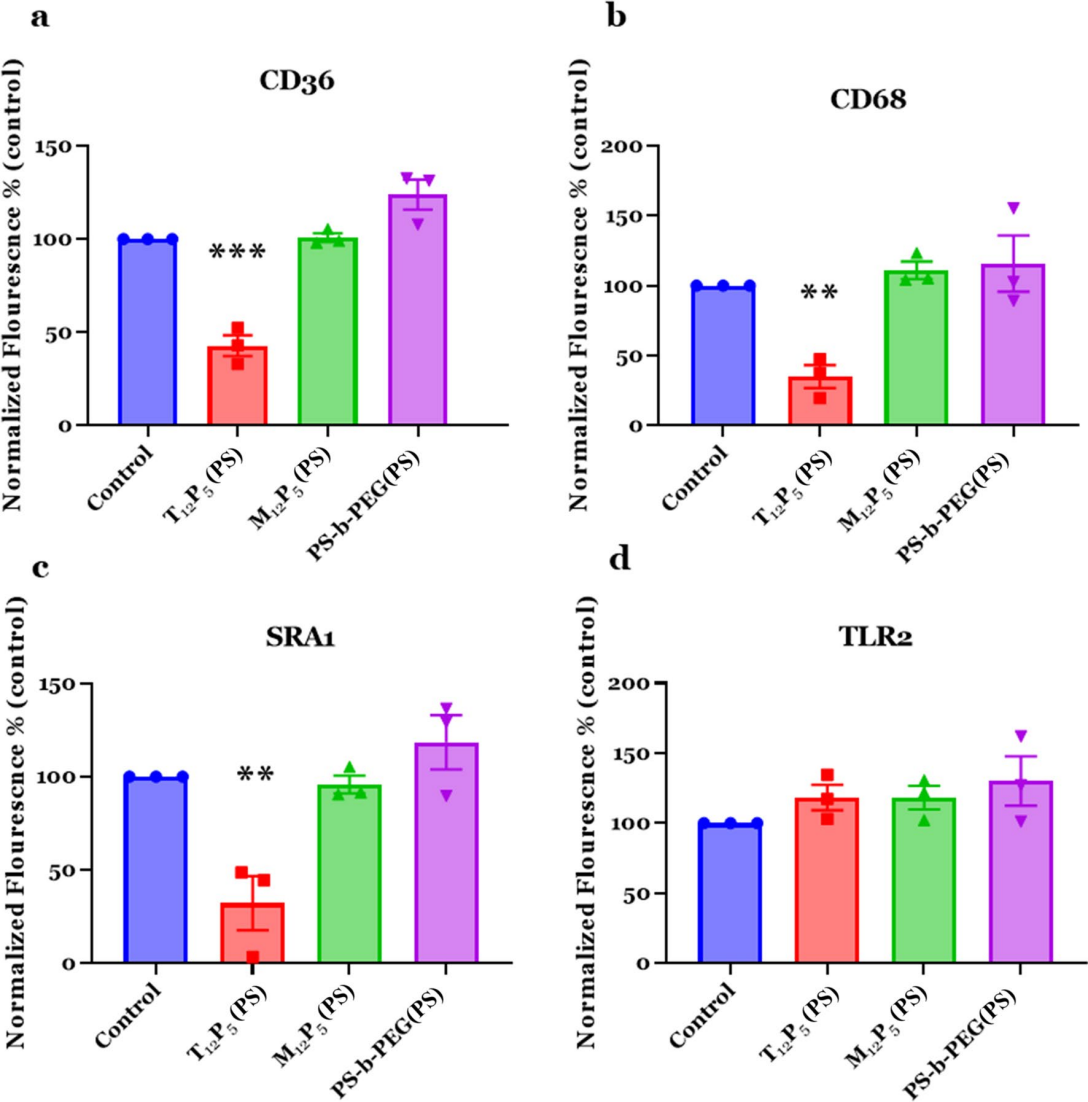
Competitive receptor binding assay in vitro. NPs reduce the surface active sites of CD36 (**a**), CD68 (**b**), SRA1 (**c**), and (**d**) TLR2 on the surface of BV2 microglia. BV2 microglia were incubated with NPs for 1 h, then co-incubated with SR-specific antibody or isotype controls for 30 min. After fixation, cells were washed, then blocked with 2% goat serum without PBS-T. To avoid internalization of NPs or antibodies, cells were incubated on ice until imaged. Cells were incubated with secondary antibodies including Alexa 488 or 594 for 1 h at room temperature. Data are presented as mean ± SEM; *n* = 3; ***P = 0.0001 for **a**, **P = 0.009 for **b**, **P = 0.0048 for **c**, Dunnett’s multiple comparisons shown on graph by one-way ANOVA

To investigate the role of AM-NPs in modulating fAβ trafficking in microglia through SRs, we first screened NP specificity to fAβ-specific SRs using the immortalized BV2 murine cell line as a cell model. BV2 cells have been widely used over the past 3 decades to recapitulate the inflammatory response associated with neurodegenerative disease as observed in primary microglia [72]. To date, a wide range of SRs have been identified to play a role in fAβ pathology, including CD36, CD68, SRA1, and TLRs. Our previous molecular modeling and docking approaches have confirmed the specificity of AM shells T_12_P_5_ and M_12_P_5_ to SRs such as CD36 and SRA1 [48, 73]. Using a cell-based competitive receptor-binding assay, we observed that NPs with tartaric acid-derived shell (T_12_P_5_(PS)) compete with antibodies for CD36, CD68, and SRA1 and significantly reduced the surface fluorescence of these receptor (*P* = 0.0001, 0.009, and 0.0048, respectively) (Fig. 3a–c). However, these NPs did not exhibit receptor-binding activity to TLR2 in this cell line (Fig. 3d). On the other hand, NPs with mucic acid-derived shell (M_12_P_5_(PS)) and control NPs (PS-*b*-PEG(PS)) did not exhibit appreciable specificity to any of the screened SRs in this cell-based assay.

**Fig. 4:**
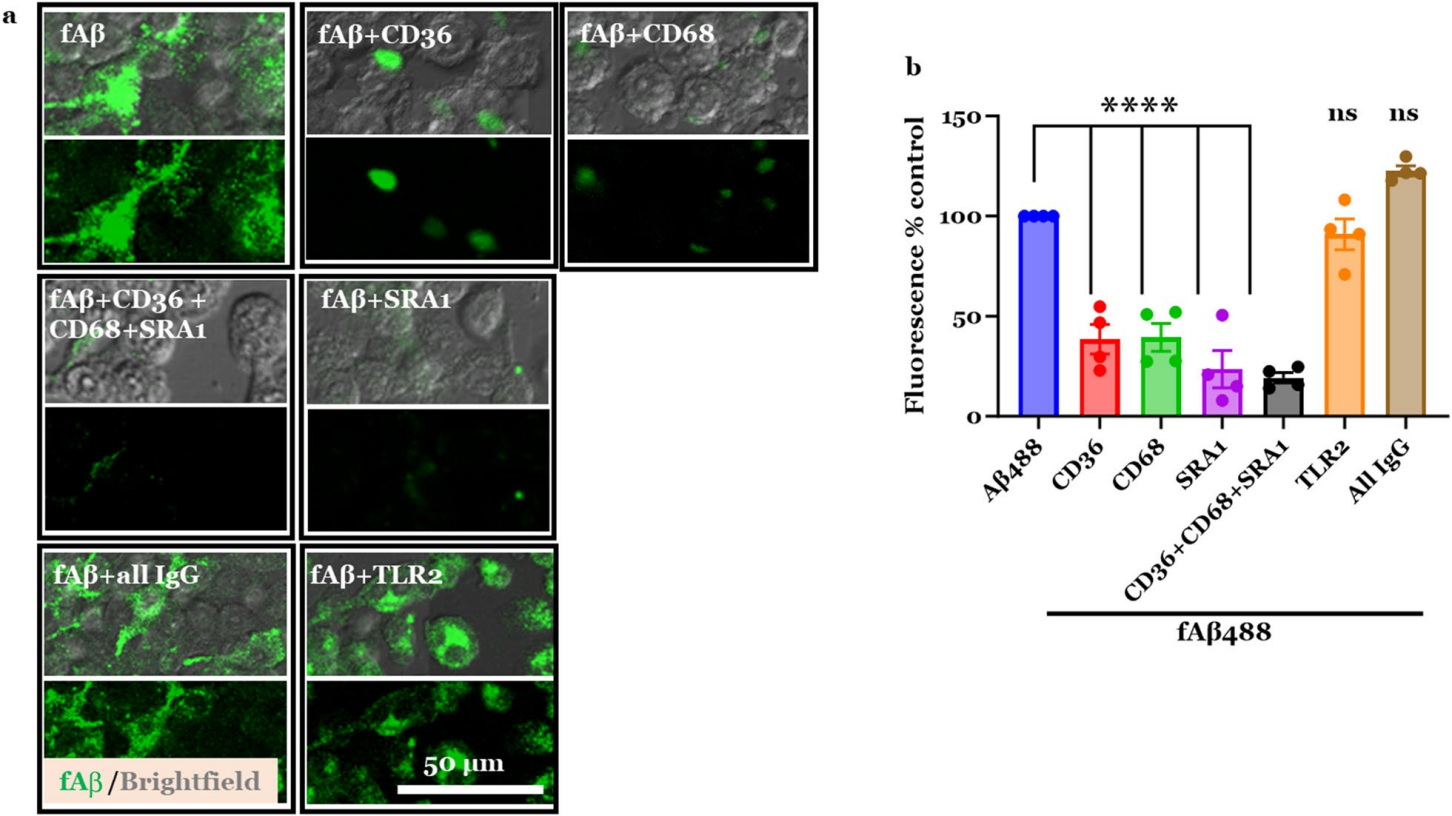
Scavenger receptors interrupt the microglial internalization of fibril amyloid beta (fAβ). BV2 microglia were treated with antibodies against scavenger receptors CD36, CD68, TLR2, and SRA1 or isotype control for 24 h, and then co-incubated with Alexa fluor 488-labeled fAβ for 24 h. The cells were fixed with 4% PFA, then washed two times with PBS to remove the extracellular fAβ. Cells were then incubated with 0.5% Triton-X-100 in potassium buffered saline (PBS-T) to remove any membrane-bound fAβ particles. **a** Representative confocal images of intracellular fAβ488 (green) and brightfield illuminated cells (grey). Scale bar, 50 µm. **b** Quantitative measurement of the intracellular Aβ488 in BV2 cells. Data are presented as mean ± SEM; *n* = 4; ****P < 0.0001 for Dunnett’s multiple comparisons shown on graph by one-way ANOVA

Next, we investigated the possible role of the SRs screened above in mediating the microglial internalization of fAβ. Cells were co-incubated with fAβ and Alexa-labeled fAβ488 in the presence or absence of receptor-specific full-length antibodies or their isotype controls at 37 °C for 24 h (Fig. 4). The fluorescence of fAβ488 was quantified and compared to the control cells treated with fAβ488 only (Fig. 4a, b). Cells blocked with CD36, CD68, or SRA1 receptor-specific antibody showed decreased internalization of fAβ (38%, 39%, and 23% of control, respectively) (*P* < 0.0001) (Fig. 4b). This observation was confirmed in cells treated with a mixture of the three receptor-specific antibodies, which exerted an additional inhibitory effect on fAβ internalization (*P* < 0.0001). These data indicated that SRs, including CD36, CD68 and SRA1, but not TLR2, are essential for the internalization of fAβ. Therefore, targeting these SRs, singly or in combination, can be a potential approach to interrupt the fAβ-mediated pathology.

**Fig. 5:**
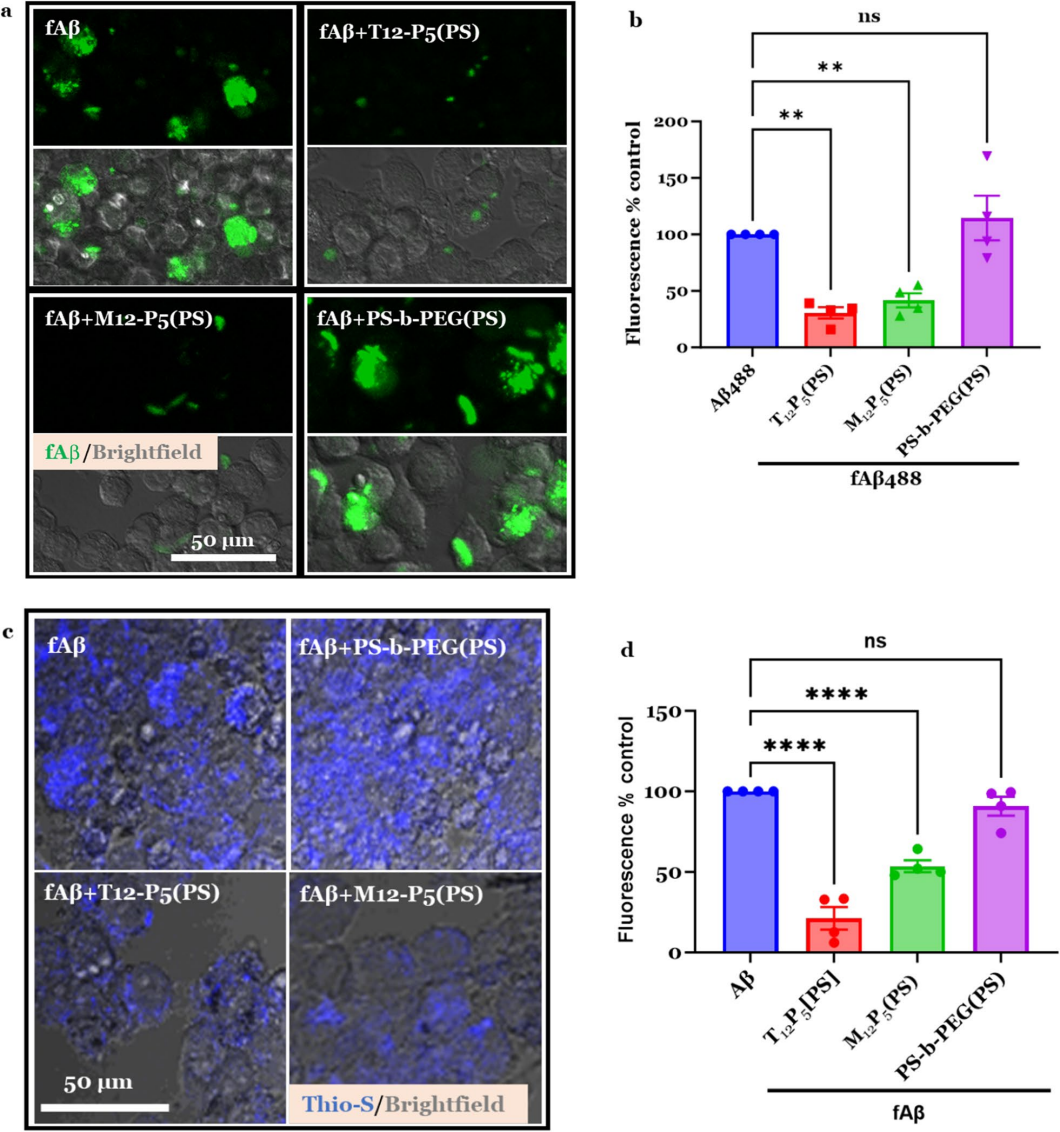
Effect of NPs on amyloid beta (Aβ) cellular uptake. BV2 microglia were treated with NPs  for 24 h and then co-incubated with Alexa fluor 488-labeled fAβ for 24 h. Cells were fixed with 4% PFA and then washed two times with PBS to remove extracellular fAβ. Cells were then incubated with 0.5% Triton-X-100 in potassium buffered saline (PBS-T) to remove any membrane-bound fAβ particles. **a** Confocal images of intracellular Alexa fluor 488-labeled fAβ (green) and brightfield illuminated cells. Scale bar, 50 µm. **b** Quantitative measurement of the intracellular fluorescence of Aβ488 in BV2 microglia. Data are presented as mean ± SEM; *n* = 4; **P = 0.0016 for T_12_P_5_ (PS) versus fAβ488, and **P = 0.0059 for M_12_P_5_ (PS) versus fAβ488, for Dunnett’s multiple comparisons shown on graph by one-way ANOVA. **c** Thioflavin-S stain (blue) of fAβ in BV2 microglia (brightfield/grey) treated with NPs for 24 h, and then co-incubated with fAβ for 24 h. **d** Quantitative measurement of the intracellular fluorescence of Thioflavin-S stain in BV2 microglia. Data are presented as mean ± SEM; *n* = 4; ****P < 0.0001 for T_12_P_5_(PS) versus fAβ488, and ****P < 0.0001 for M_12_P_5_(PS) versus fAβ488, for Dunnett’s multiple comparisons shown on graph by one-way ANOVA

Next, given the molecular modeling and the in vitro results shown earlier, we tested the hypothesis that NPs can interrupt the microglial uptake of fAβ. Cells were co-incubated with fAβ and Alexa-labeled fAβ488 in the presence or absence of AM-NPs or their NP control (PS-*b*-PEG(PS)) at 37 ºC for 24 h (Fig. 5, Additional file 1: Fig. S1). The fluorescence of fAβ488 was quantified and compared to the control cells treated with fAβ488 only (Fig. 5a, b). The fluorescence of fAβ488 was markedly reduced by 70% and 60% in cells treated with NPs with bioactive shells T_12_P_5_ (*P* = 0.0016) and M_12_P_5_ (*P* = 0.0059) compared to control cells treated with fAβ488 (Fig. 5b). This reduction in fAβ is presumably due to the putative binding ability of the bioactive shell T_12_P_5_ to SRs on the microglial surface. This was confirmed by the observation that the fluorescence of fAβ488 in cells treated with control NPs comprising of the inactive shell (PS-*b*-PEG) was not altered compared to control cells treated with fAβ488 only. To further validate this result, we used Thioflavin-S stain to label the intracellular beta-sheets of fibrils. BV2 microglia were co-incubated with 20 mM of fAβ for 24 h after pre-incubation in the presence or absence of AM-NPs for 24 h. Cells were stained with 0.01% Thioflavin-S stain for 30 min. The fluorescence of thioflavin-S was quantified and compared to the control cells treated with fAβ only (Fig. 5c, d). Consistently, the intracellular fAβ was significantly reduced by 79% and 48% in cells treated with NPs with bioactive shells T_12_P_5_ (*P* < 0.0001) and M_12_P_5_ (*P* < 0.0001), respectively, compared to control cells treated with fAβ488 (Fig. 5c, d). Collectively, these results confirm the ability of AM-NPs to interrupt microglial fAβ uptake through combined effects on fAβ-specific SRs.

### AM-NPs modulate microglial inflammatory response and neurotoxicity

**Fig. 6:**
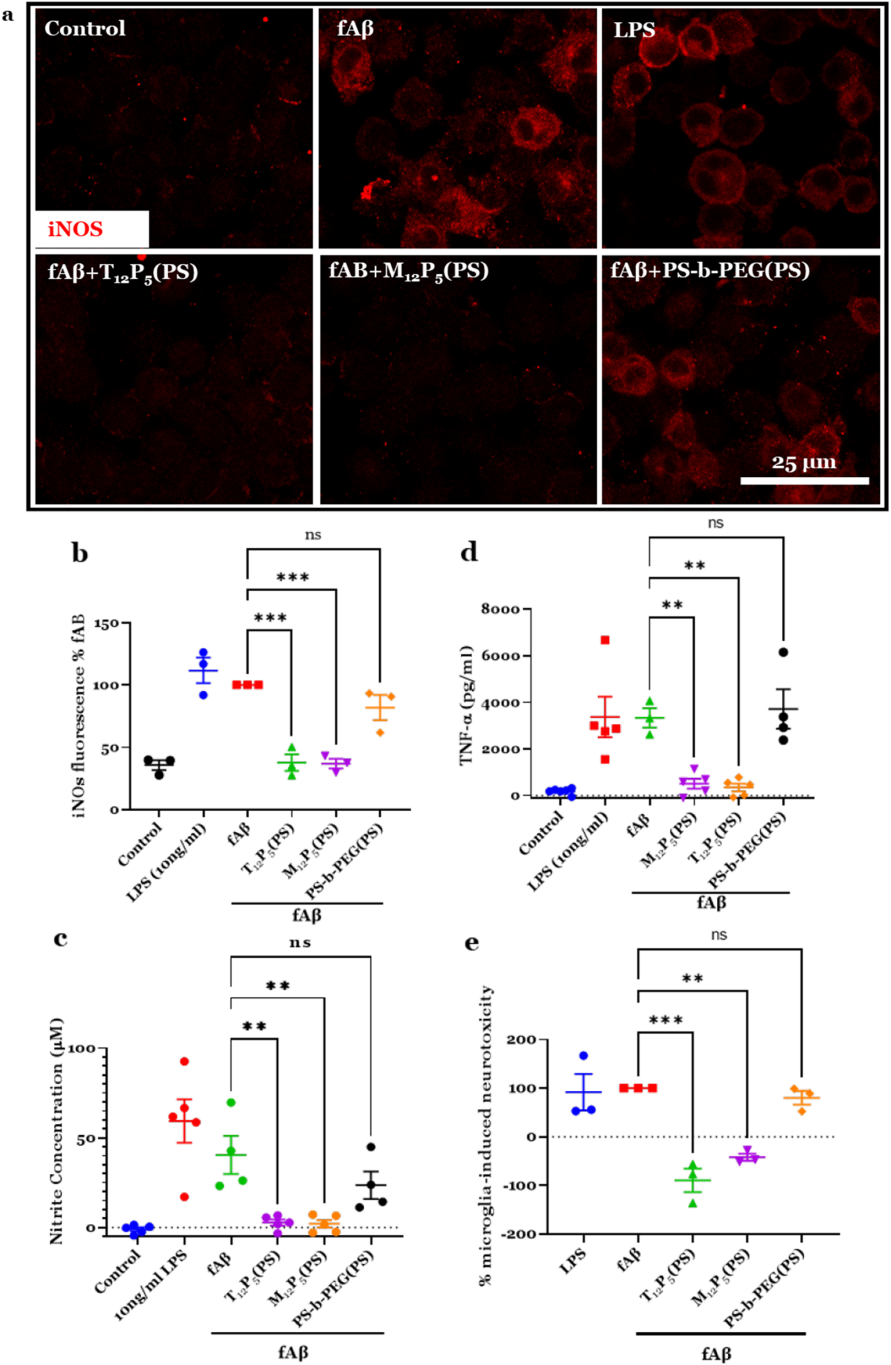
AM-NPs suppress Aβ-mediated microglial activation. **a**–**c** BV2 microglia were treated with 20 µM Aβ in the presence or absence of NPs or 10 ng/ml lipopolysaccharide (LPS). After 24 h, supernatants were collected and assayed for inflammatory markers. Cells were fixed with 4% PFA and then permeabilized in 0.5% Triton-X-100 in potassium-buffered saline (PBS-T). After blocking, cells were incubated with anti-iNOS antibody overnight at 4 °C, then washed with PBS-T. Cells were incubated with secondary antibody Alex 594 for 1 h. **a** Confocal images representing the expression of inducible nitric oxide synthase (iNOS) in BV2 cells after 24-h co-incubation with fAβ and NPs. Scale bar, 25 µm. **b** Quantitative analysis of fluorescence intensity of iNOS in BV2 cells after 24-h co-incubation with fAβ and NPs. Data are presented as mean ± SEM; *n* = 3; ***P = 0.0002 for T_12_P_5_ versus fAβ, ***P = 0.0001 for M_12_P_5_ versus fAβ for Dunnett’s multiple comparisons shown on graph by one-way ANOVA.** c** Nitrite concentration measured by Griess reagent in the conditioned media harvested 24 h after cell co-incubation with Aβ and NPs. Data are presented as mean ± SEM; *n* = 3; **P = 0.0057 for T_12_P_5_ versus fAβ, **P = 0.0048 for M_12_P_5_ versus fAβ for Dunnett’s multiple comparisons shown on graph by one-way ANOVA. **d** Concentration of TNFα measured by ELISA in the conditioned media harvested 24 h after cell co-incubation with fAβ and NPs. Data are presented as mean ± SEM; *n* = 3; **P = 0.042 for T_12_P_5_ versus fAβ, **P = 0.0068 for M_12_P_5_ versus fAβ for Dunnett’s multiple comparisons shown on graph by one-way ANOVA. **e** SH-SY5Y cells were treated with conditioned media of  fAβ- or LPS-stimulated BV2 cells in the presence or absence of NPs. Cytotoxicity in response to stimulated BV2 conditioned media was quantified in SH-SY5Y media using LDH assay. Data are presented as mean ± SEM; *n* = 3; ***P = 0.003 for T_12_P_5_ versus fAβ and **P = 0.0027 for M_12_P_5_ versus fAβ for Dunnett’s multiple comparisons shown on graph by one-way ANOVA

The recognition of fAβ by SRs on microglia results in a pro-inflammatory response characterized by the release of pro-inflammatory cytokines and chemokines. Excessive exposure to fAβ results in microglial activation and a shift from quiescent to a pro-inflammatory microglial phenotype. Therefore, we studied the effect of AM-NPs on fAβ-mediated pro-inflammatory response in microglia via assessing the cellular expression of iNOS and the levels of released TNF-α and NO (Fig. 6). BV2 microglia treated with fAβ were co-incubated with or without AM-NPs for 24 h. Cellular expression of iNOS was significantly reduced in fAβ-stimulated cells co-incubated with T_12_P_5_(PS) (*P* = 0.0002) or M_12_P_5_(PS) (*P* = 0.0001) compared to control cells treated with fAβ only (Fig. 6a, b). In line with this result, the concentration of NO released from fAβ-stimulated microglia co-incubated with T_12_P_5_(PS) (*P* = 0.0057) or M_12_P_5_(PS) (*P* = 0.0048) was significantly reduced compared to control cells treated with fAβ only (Fig. 6c). Similarly, the release of TNF-α was significantly reduced in the condition of co-incubation with T_12_P_5_(PS) (*P* = 0.0042) or M_12_P_5_(PS) (*P* = 0.0068) compared to control cells treated with fAβ only (Fig. 6d). Given the pronounced inflammatory response in fAβ-stimulated cells co-incubated with PS-*b*-PEG(PS) NPs, our results confirm the anti-inflammatory effects of the bioactive shells T_12_P_5_ and M_12_P_5_ on fAβ-mediated activation.

In AD, microglial activation and the subsequent release of pro-inflammatory cytokines lead to neuronal damage and loss of neuronal circuits [19, 74]. Therefore, given the anti-inflammatory effects of AM shells described earlier, we investigated the role of AM-NPs in modulating microglia-mediated neurotoxicity. To recapitulate the cell interplay in AD brain, we employed a conditioned media approach based on BV2 microglial and SH-SY5Y neuroblastoma cells. fAβ-stimulated BV2 cells were co-incubated with or without AM-NPs T_12_P_5_(PS), M_12_P_5_(PS), or PS-*b*-PEG(PS) for 24 h. In parallel, positive control cells were treated with LPS for 24 h. SH-SY5Y cells were then treated with the harvested BV2 CM and incubated for 24 h. Treatment with T_12_P_5_(PS) or M_12_P_5_(PS) CM significantly reduced neurotoxicity (*P* = 0.003 and 0.0027, respectively) (Fig. 6e). Consistent with the microglial activation studies, the non-bioactive shell of control NP PS-*b*-PEG(PS) did not counteract the BV2-mediated neurotoxicity, which supports our hypothesis that the bioactive shells can modulate the neurotoxicity mediated by activated microglia.

### AM-NPs modulate NF-kB nuclear translocation and induce lysosomal clearance

**Fig. 7:**
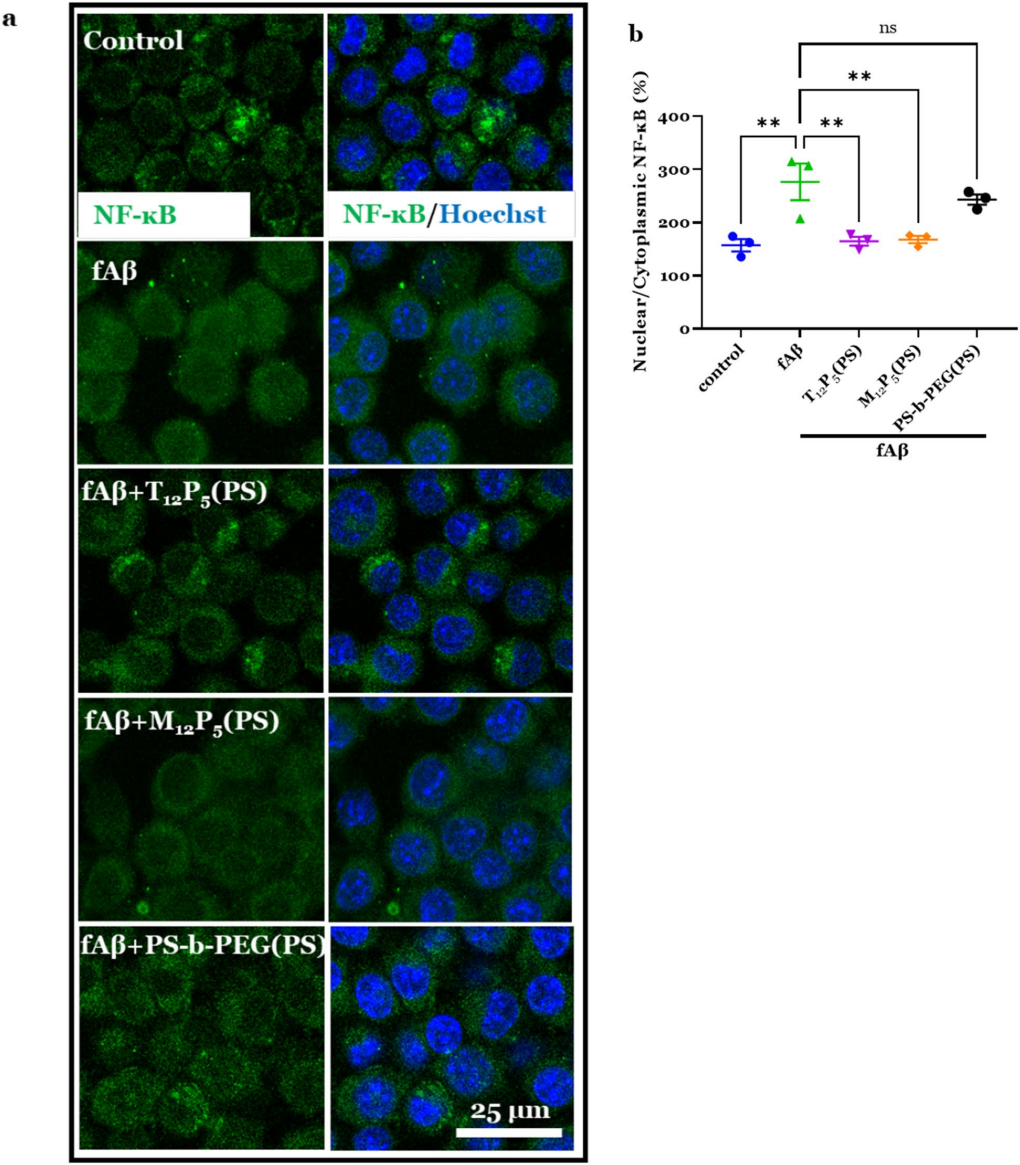
NPs mediate fAβ-induced NF-ĸB nuclear translocation in microglia. **a**, **b** BV2 microglia were treated with 20 µM fAβ with or without NPs for 2 h. Cells were immediately fixed with 4% PFA. Cells were then blocked using 2% blocking buffer, incubated with anti-NF-ĸB P65 antibody overnight at 4 °C, and washed with PBS-T. Cells were incubated with secondary antibody Alex 488 for 1 h and nuclei were counterstained with Hoechst (blue). **a** Representative confocal microscopy images showing NF-ĸB nuclear translocation. Scale bar, 25 µm. **b** Quantitative measurement of the nuclear translocation of NF-ĸB in BV2 cells treated with Aβ with or without NPs for 2 h. Data are presented as mean ± SEM; *n* = 3; **P = 0.0027 for fAβ versus control, **P = 0.0044 for T_12_P_5_ versus fAβ and **P = 0.0054 for M_12_P_5_ versus fAβ, for Dunnett’s multiple comparisons shown on graph by one-way ANOVA

The production of inflammatory cytokines and chemokines is regulated by upstream events including translocation of cytoplasmic NF-kB into the nucleus. In quiescent microglia, NF-kB is sequestered in the cytoplasm; however, following a cellular stimulus such as fAβ, NF-kB is translocated into the nucleus where it induces the expression of pro-inflammatory cytokines and chemokines genes [75, 76]. Given the modulatory effects of AM-NPs on fAβ-mediated inflammation, we examined whether these effects are due to a regulatory effect of NPs on an upstream event such as NF-kB nuclear translocation. fAβ-stimulated BV2 microglia were co-incubated with or without AM-NPs for 2 h, and then cells were immediately fixed. Using immunocytochemistry and confocal microscopy, the immunoreactivities of the nuclear and the cytoplasmic NF-kB were determined in cells counterstained with Hoechst (Fig. 7a, b). Treatment with fAβ significantly increased the nuclear NF-kB (*P* < 0.0027) (Fig. 7b). Strikingly, the nuclear NF-kB was significantly reduced in fAβ-stimulated cells co-incubated with T_12_P_5_(PS) (*P* < 0.0044) or M_12_P_5_(PS) (*P* < 0.0054), which attenuated the fAβ-induced inflammatory response.

**Fig. 8:**
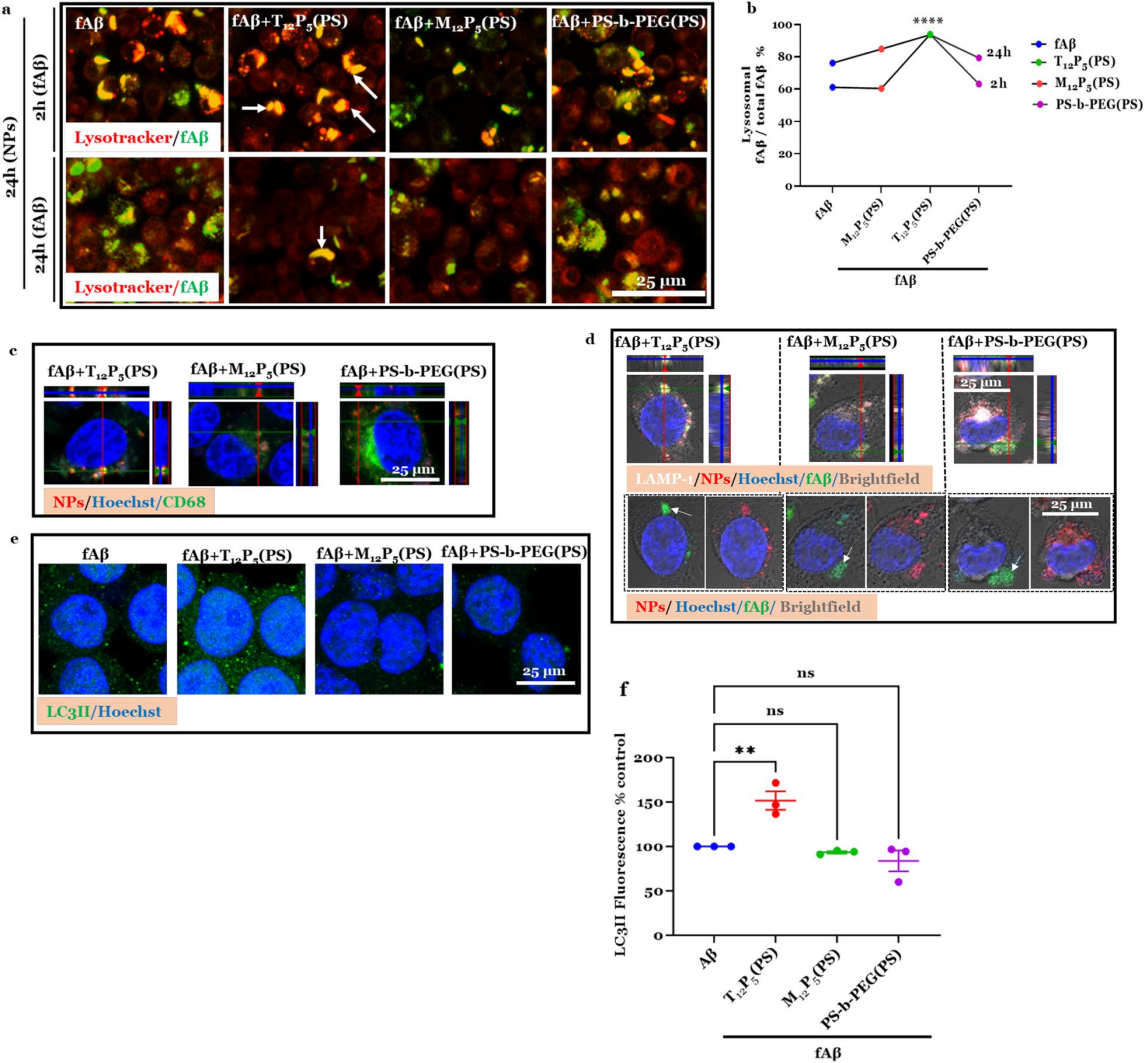
AM-NPs accelerate lysosomal clearance of fAβ. **a**–**d** BV2 microglia were incubated with NPs for 24 h and then co-incubated with Alexa Fluor 488-labeled fAβ for 2 h or 24 h. Lysosomes in treated live BV2 microglia were stained with 70 µM Lysotracker (red) for 30 min. Cells were then fixed using 4% PFA. **a** Representative confocal microscopy images showing fAβ-positive (green) lysosomes (red). White arrows show fAβ in lysosomes. Scale bar, 25 µm. **b** Quantitative measurement of the lysosomal degradation of intracellular Aβ at 2 h and 24 h in BV2 microglia pretreated with NPs. Data are presented as mean ± SEM; *n* = 3; ****P < 0.0001 for T_12_P_5_ versus fAβ, for Dunnett’s multiple comparisons shown on graph by two-way ANOVA. **c**, **d** BV2 microglia incubated with NPs for 24 h then co-incubated with Alexa Fluor 488-labeled fAβ for 2 h. Cells were fixed with 4% PFA, permeabilized using PBS-T, and then blocked using 2% goat blocking buffer. Cells were then incubated with anti-CD68 or LAMP-1 antibody overnight at 4 °C, then washed with PBS-T. Cells were incubated with secondary antibody Alex 488 or 954 for 1 h and nuclei were counterstained with Hoechst. **c** Orthogonal projection of confocal Z-stacks shows labeling of CD68 (green), Dil-labeled NPs (red), and nuclear stain Hoechst (blue). **d** Orthogonal projection of confocal Z-stacks shows labeling of LAMP-1 (white), Dil-labeled NPs (red), nuclear stain Hoechst (blue), and fAβ488 (green) in brightfield illuminated cells in the top panel, and co-labeling of nuclear stain Hoechst (blue), Dil-labeled NPs (red), and fAβ488 (green) in brightfield illuminated cells in the bottom panel. White arrows show intracellular fAβ. **e**, **f** BV2 microglia were incubated with NPs for 24 h and then co-incubated with fAβ for 30 min. **e** Confocal images of LC3 immunoreactivity (green) in BV2 microglia co-stained with Hoechst (blue). **f** Quantitative analysis of LC3 fluorescence intensity in BV2 microglia. Data are presented as mean ± SEM; *n* = 3; **P < 0.01 for T_12_P_5_ versus fAβ, for Dunnett’s multiple comparisons shown on graph by two-way ANOVA. Scale bar, 25 µm

**Fig. 9:**
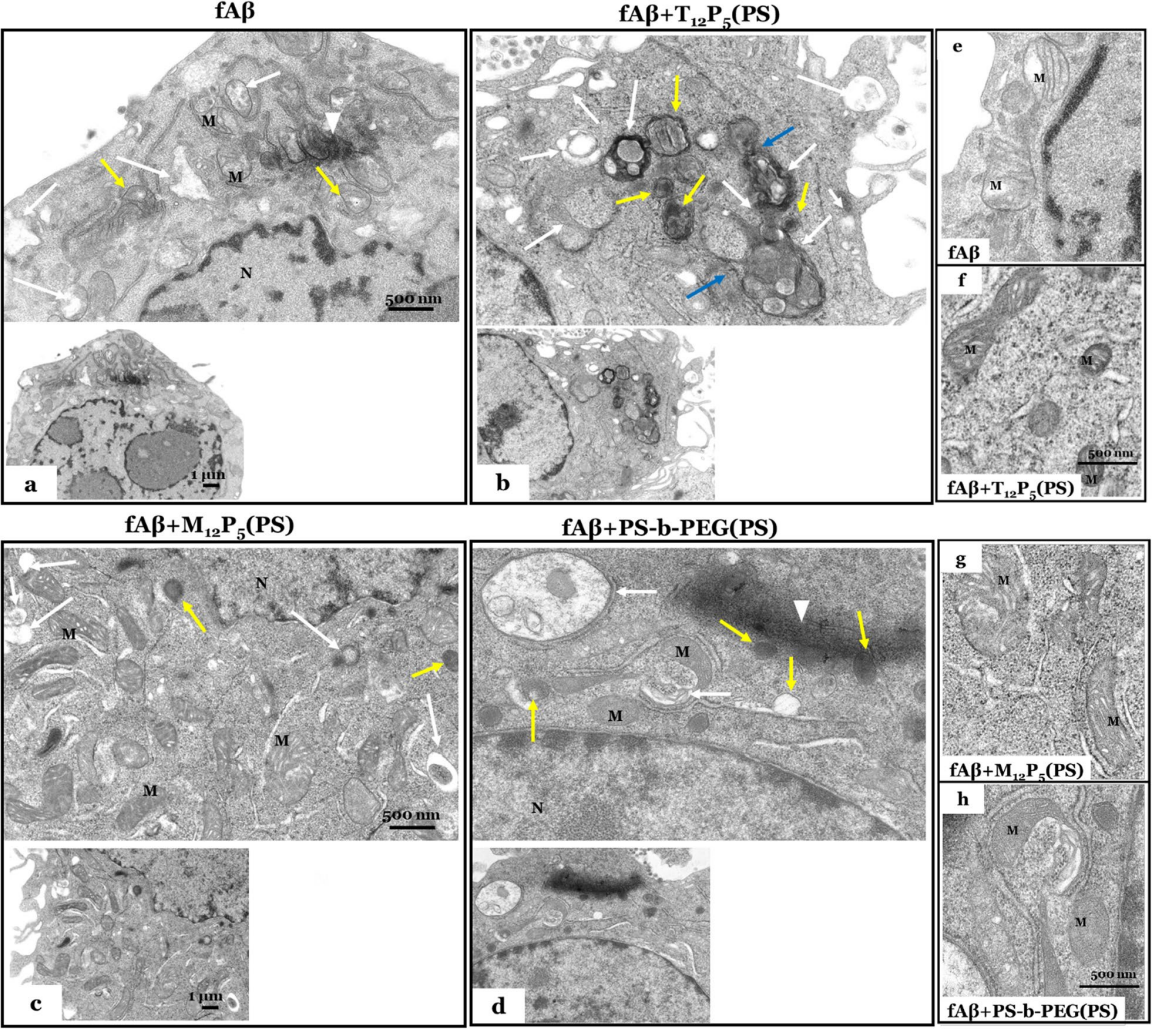
AM-NPs accelerate clearance of fAβ. **a**–**d** Electron microscope micrographs of BV2 microglia incubated with NPs for 24 h, then co-incubated with fAβ for 30 min. Cells were fixed with a mixture of 2.5% glutaraldehyde and 4% PFA in 0.1 M cacodylate buffer at pH 7.4. Ultrathin (90 nm) sections of cell pellets were imaged using the electron microscope. **a**, **e** The ultrastructure of a microglia treated with fAβ only. The intracellular fAβ (white arrowhead) is surrounded by damaged vacuoles, including large autophagosomes (white arrows) and lysosomes with incomplete membranes (yellow arrows). Mitochondria (M) in this cell is structurally damaged, as evidenced by disordered cristae and a thin outer membrane (**e**). **b**, **f** The ultrastructure of a fAβ-treated microglia co-incubated with T_12_P_5_(PS). In this cell, the phagocytic activity is high, as illustrated by the high representation of a variety of autophagosomes (white arrows), lysosomes with well-developed membranes (yellow arrows), and autophagosome-lysosome fusion structures (blue arrows). Mitochondria in this cell are intact, as evidenced by well-defined cristae structures and an intact outer membrane (**f**). **c**, **g** The ultrastructure of fAβ-treated microglia co-incubated with M_12_P_5_(PS). The phagocytic activity is less, as illustrated by fewer autophagosomes (white arrows); however, the lysosomal structures are characterized by a well-developed double membrane (yellow arrows). This cell has a high representation of intact and long mitochondria characterized by well-defined cristae and an intact outer membrane (**g**). **d**, **h** The ultrastructure of fAβ-treated microglia co-incubated with the control PS-*b*-PEG(PS). A large center of fAβ is present in this cell, along with large thin-walled autophagosomes (white arrows) and thin-walled lysosomal structures (yellow arrows). Mitochondria in this cell are structurally damaged, as evidenced by distorted cristae and thin outer membrane. N, nucleus, and M, mitochondria. Scale bars, 500 nm in the top panels of **a**–**d** and **e**–**h**; 1 µm in the bottom panels in **a**–**d**

Chronic activation of microglia due to prolonged exposure to fAβ results in a loss of homeostatic microglial function and imbalanced clearance of fAβ. Dysregulation of fAβ clearance, which has been observed in the brains of AD patients and animal models [77–79], exacerbates fAβ burden and ultimately leads to neuronal damage. As our data revealed modulatory effects of AM-NPs on early events of the fAβ pathway, including Aβ fibrilization, cellular uptake, NF-kB nuclear translocation, and microglial activation, we investigated the possible effect of NPs on the lysosomal function related to clearance in microglia. BV2 microglia were co-incubated with fAβ for either 2 h or 24 h after pre-incubation for 24 h with or without AM-NPs. fAβ in cells treated with T_12_P_5_(PS) was significantly (*P* < 0.0001) localized in lysosomes compared to control cells treated with fAβ only (Fig. 8a, b), while fAβ in cells co-treated with the control NPs did not show any change from the control cells treated with fAβ only. To further validate the observation that T_12_P_5_ accelerated the degradation of fAβ at 2 h, immunoreactivities of LAMP-1 and CD68 were assayed. Using colocalization approaches, the internalized fluorescently labeled T_12_P_5_(PS) demonstrated a high degree of colocalization with fAβ in lysosomes as evidenced by colocalization with LAMP-1 (Fig. 8d). These results were confirmed by the observation that the fluorescently labeled T_12_P_5_(PS) was shown to be colocalized with the lysosomal receptor CD68 (Fig. 8c). Interestingly, in cells treated with M_12_P_5_(PS), fAβ488 was found in association with the fluorescently labeled NPs; however, these molecules were not in association with LAMP-1. On the other hand, in cells treated with control NPs, the fluorescently labeled NPs were diffused in most areas of the cytoplasm, while fAβ488 was observed in LAMP-1-negative compartment in the cytoplasm (Fig. 8d), indicating the inefficient ability of the lysosomal clearance of fAβ deposits in the cell. The observation that T_12_P_5_(PS) was colocalized with the lysosomal receptor CD68 (Fig. 8c) confirmed the hypothesis that these NPs may accelerate fAβ clearance through specific binding and recognition of CD68 that may act as a cargo molecule between the cytoplasm and the lysosome [80]. To further validate our hypothesis, we investigated the effect of our NPs on the autophagic activity, a key upstream event of fAβ lysosomal degradation, in fAβ-activated microglia [81, 82]. BV2 cells were incubated with NPs for 24 h and then co-treated with fAβ for 30 min. The immunoreactivity of the autophagic marker microtubule-associated protein 1A/1B-light chain 3 II (LC3II) was assayed (Fig. 8e, f). Our results showed significant activation of LC3II in the fAβ-stimulated cells co-incubated with T_12_P_5_(PS) (*P* = 0.0045) compared to control cells treated with fAβ only (Fig. 8e, f). Consistent with our findings on the lysosomal activity, cells co-treated with M_12_P_5_(PS) and the control NPs did not show significant LC3II activity. We further investigated the ultrastructure of those BV2 cells using electron microscopy (Fig. 9). The phagocytic activity of the fAβ-stimulated cells was interrupted as evidenced by the presence of a core center of fAβ surrounded by damaged vacuoles including larger autophagosomes, and thin-walled lysosomes (Fig. 9a). Notably, mitochondria in those cells were structurally damaged as evidenced by their disordered cristae and a thin outer membrane (Fig. 9e). In contrast, the T_12_P_5_(PS)-treated cells showed high phagocytic activity. Those cells were characterized by the high representation of a variety of well-enveloped autophagosomes, lysosomes with well-developed membranes, and autophagosome-lysosome fusion structures (Fig. 9b). Consistently, the mitochondria in these cells were intact, as evidenced by well-defined cristae structures and an intact outer membrane (Fig. 9f). In the M_12_P_5_(PS)-treated cells, the phagocytic activity was not as striking as that in T_12_P_5_(PS)-treated cells; however, there was no sign of fAβ core centers in those cells (Fig. 9c). Given the anti-inflammatory effect of this AM and the low level of intracellular fAβ, a unique role of M_12_P_5_(PS) in clearing fAβ can be suggested. One of the possibilities is that M_12_P_5_(PS) may activate the mitochondrial engulfment of fAβ. This is evidenced by the presence of healthy elongated mitochondria in those cells (Fig. 9g). Consistent with our findings, the control NP-treated cells did not show any sign of active phagocytosis. A core center of fAβ was observed together with large thin-walled autophagosomes and thin-walled lysosomal structures (Fig. 9d). The mitochondria in these cells were structurally damaged, as evidenced by distorted cristae and thin outer membrane (Fig. 9h).

These results suggest another unique feature of the tartaric acid-based shells, possibly consistent with their interactions with lysosomal receptor CD68 as described earlier.

## Discussion

Current therapeutic strategies for AD treatment aim to either compensate for neuronal damage via cholinergic or neurotrophic factors [83, 84] or to eliminate protein aggregation [85, 86]. However, these treatments fail to halt the underlying cognitive decline and neuronal damage. The most recent FDA-approved anti-Aβ antibody drug, Lecanemab, was designed to target proto-fibrils and plaques of Aβ rather than monomeric Aβ in early and mild stages of AD [14]. This Aβ-targeting drug has shown efficiency to reduce fAβ burden while slowing the cognitive decline in early AD patients by 27% [14]. Research is underway to address its efficacy for people at risk including the first-degree relatives, and its potential ability to stabilize late-stage AD patients. Given the complexity of the disease and the involvement of several components at different stages of AD, it is plausible that an effective treatment regimen may need to address the systems-level orientation of the disease that is triggered by multifactorial events.

In this study, we hypothesized that a multimodal nanotherapeutic technology could be leveraged to target the inflammatory component of the disease while alleviating the fAβ burden and subsequent neuronal damage. We demonstrate that the sugar-based anionic AM-NPs can slow the transition of Aβ monomers to mature fibrils and reduce the amount of fAβ formed under conditions that favor fibrilization (Fig. 2). Furthermore, AM-NPs showed disaggregating effect on the preformed Aβ fibrils (Fig. 2e). This anti-amyloidosis effect of NPs may be mediated by the electrostatic and hydrophobic interactions between the anionic aliphatic AM shells and the cationic lysine residues in Aβ monomers, which have been shown to play important roles in the assembly and toxicity of Aβ and α-syn [87, 88]. Both AMs M_12_P_5_ and T_12_P_5_ are lipophilic anionic molecules composed of mucic acid and tartaric acid backbones with aliphatic side chains and acidic end groups. This interaction between AM-NPs and Aβ may lower the concentration of free monomers and shift the equilibrium away from fibrilization. Another possibility is that the interactions between AM-NPs and oAβ may be strong enough to interrupt monomer binding, slowing the nucleation and fibril formation. Under physiological pH conditions, Aβ exhibits net negative charges; however, the positively charged moieties in Aβ play a critical role in seeding and self-assembly [87]. In line with our findings, it was shown that the anionic lysine-specific molecular tweezers efficiently inhibit fibrilization of proteins, including Aβ, tau, and α-syn, through hydrophobic and electrostatic interactions [89, 90]. Furthermore, targeting the His13-Lys16 cluster region of Aβ using inorganic anionic compounds was found to inhibit Aβ aggregation through electrostatic and hydrophobic interactions [91]. Polyphenols, through hydrophobic interactions with the hydrophobic core in Aβ, are sufficient to inhibit protein aggregation, promote disaggregation of preformed Aβ fibrils, and reduce the associated cytotoxicity [92]. A previous study showed that a small polyphenol epigallocatechin-3-gallate (EGCG) that is present in green tea, can remodel fAβ through Schiff base interactions with free amines in fAβ [93]. However, this mechanism is unlikely to account for the disaggregating effect of the AM-NPs on the preformed Aβ fibrils (Fig. 2e), because the AMs we designed feature ester and carboxylic acid functional groups that do not react with Schiff bases [48]. Further studies could be done to elucidate the molecular binding mechanism between AMs and Aβ, which will aid in the development of disease-modifying therapies for amyloidosis.

Our studies demonstrated that several microglial SRs, including SRA1, CD36, and CD68, mediate fAβ internalization (Fig. 4). Although a growing body of evidence supports the role of CD36 and SRA1 in fAβ-mediated pathology [33, 94–96], little is known about CD68 as a key scavenger receptor mediating fAβ trafficking. To our knowledge, no studies have investigated the mechanistic role of CD68 in AD. Only two studies with controversial findings were focused on the microglial expression of CD68 in AD patients after Aβ immunization [97, 98]. Our findings provide proof-of-concept about the involvement of CD68 in the trafficking of fAβ into microglia. CD68 is the only known member of the class D scavenger receptors and has been widely exploited as a macrophage marker. It belongs to the LAMP family and is located mainly in the lysosomal membrane but can rapidly shuttle to the cell surface where it binds the oxidized low-density lipoproteins, apoptotic cells, and phosphatidylserine [80, 99, 100]. Here, the tartaric acid-derived AM-NPs were shown to competitively bind SRA1, CD36, and CD68 in the presence of receptor-targeting antibodies. The NP T_12_P_5_(PS) could efficiently block CD68 and inhibit its binding with anti-CD68 antibody by 75%.

We have previously validated the interactions of T_12_P_5_ with CD36 and M_12_P_5_ with CD36 and SRA1 receptors [48, 73]. This study extended our knowledge about T_12_P_5_ to include SRA1 and CD68 as receptor-specific macromolecules. Treatment with T_12_P_5_ alone was sufficient to interrupt the internalization of fAβ into microglia to an extent comparable to that elicited by combined antibodies for receptors SRA1, CD36, and CD68 (Fig. 5). In line with our findings, SRA1 deficiency causes a 50% reduction in Aβ uptake by microglia in AD transgenic mice [35]. Also, fAβ uptake by microglia isolated from SRA1-knockout mice was reduced by 60%; however, other SRs ligands were shown to prevent fAβ uptake in those cells [36]. These results indicate that other SRs may be involved in the uptake of fAβ. Of these receptors, SR-B, a member of the CD36 superfamily, has been widely studied in fAβ trafficking and microglial activation [30, 95, 96]. Despite the findings that the M_12_P_5_ shells had no competing effects with SR-specific antibodies, these molecules significantly interrupted fAβ uptake by microglia (Fig. 5). This finding is consistent with previous studies, which reported that the M_12_P_5_ shells bind SRA1 and CD36 [73] and markedly interrupt the internalization of α-syn into microglia [49]. Key differences between these AMs and SR antibodies include lipophilicity, charge, and binding sites on SRs. It is possible that M_12_P_5_ AMs block other intracellular epitopes on SRs that bind to and facilitate fAβ uptake. Also, it is possible that these AMs may be able to prevent the formation of multi-receptor complexes that exert a signaling effect in the progression of fAβ pathogenesis. It has been reported that CD36 induces microglial pro-inflammatory signaling through heterodimer assembly of TLR2 and TLR4 [101]. Moreover, interrupting the complex formed by CD36 receptor, integrin-associated protein CD47 and α6β1-integrin prevents cell adhesion to fAβ and the subsequent pro-inflammatory response [102]. Further investigations are warranted to elucidate the nature of interactions between these AMs and SRs.

While many small molecules have been shown to modulate microglial activation and attenuate the inflammatory response, little is known about their stability to ensure continued bioactivity and specificity to microglia. In this study, we investigated the role of AM-NPs in inhibiting fAβ-mediated microglial activation and inflammatory response. Given their SR-specificity (Fig. 3) [48, 73] and anti-amyloidosis effects (Fig. 2c, d), we hypothesized that the AM-NPs may play a role in mediating the microglial phenotype. The AM-NPs were shown to interrupt fAβ-mediated microglial activation, pro-inflammatory response, and neurotoxicity (Fig. 6); they significantly reduced the levels of iNOS, TNF-α and NO to the baseline level in cells stimulated with fAβ. Because SRA1 and CD36 are both shown to mediate microglial fAβ uptake and consequently pro-inflammatory cytokine secretion [94, 101], it is possible that the combined inhibitory effect of AM-NPs on multiple SRs account for the observed anti-inflammatory effects in the presence of fAβ. An earlier study showed that the CD36-blocking antibodies reduced the secretion of ROS by 50% from cells plated on fAβ-coated surfaces [96]. Consistent with this observation, CD36 engagement by fAβ initiates a CD36-dependent signaling cascade, which leads to an inflammatory response, including the production of ROS and chemokines. Inhibition of this signaling cascade was found to significantly reduce the level of ROS in the presence of CD36 and fAβ in vitro [103]. Similar results were observed in CD36-knockout macrophages stimulated by fAβ [34]. These results indicated that SRs and the downstream signaling cascade are essential to fAβ-mediated inflammatory response. In our study, unlike antibody treatments, the AM-NPs showed multi-faceted mechanisms in reducing intracellular fibrils, the associated pro-inflammatory response, and neurotoxicity. Therefore, targeting multiple SRs using AM-NPs is a multi-faceted approach to interrupting fAβ uptake and the downstream signaling events essential for fAβ pathological progression.

NF-κB is a well-established transcription factor that plays a central role in mediating the inflammatory response in AD [104]. Under physiological conditions, NF-κB is sequestered in the cytoplasm; however, upon exposure to pro-inflammatory stimuli, it translocates to the nucleus. The nuclear translocation of NF-κB activates the expression of pro-inflammatory cytokines and chemokines [105]. Our results showed that T_12_P_5_(PS) and M_12_P_5_(PS) could significantly reduce the fAβ-induced nuclear translocation of NF-κB (Fig. 7). This finding indicates that the NF-κB signaling pathway may be the mechanism underlying the AM-NP modulation of fAβ-induced microglial activation and the pro-inflammatory response. Since the extensive release of pro-inflammatory cytokines can activate the NF-κB signaling pathway, it is possible that AM-NPs potentially interrupt this vicious cycle of fAβ-mediated microglial activation. Molecules that target the NF-κB signaling pathway have been shown to decrease inflammatory response, improve behavioral deficits, and promote neuroprotection in AD models [106–108]. NF-κB signaling components have been identified as the top upstream regulators in tau-stimulated microglia. For example, inhibition of the NF-κB pathway by TPCA-1 was found to reduce the release of phosphorylated tau from microglia and rescue tau-associated learning and memory deficits [109]. The AM-NPs could provide insights into the development of a mechanism-based therapeutic to target NF-κB translocation in multiple cell types and arrest microglial activation in several neurological disorders.

Microglia, as the native immune cells in the CNS, are critical components for lysosomal clearance of misfolded Aβ. However, lysosomal degradation of fAβ has been shown to be interrupted in AD [77, 85, 110]. The key AM composition identified in our study, T_12_P_5_, promoted acute fAβ lysosomal localization over 2-h and 24-h courses of fAβ treatment (Fig. 8). It is possible that the specific interaction of T_12_P_5_ with the lysosomal CD68 (Fig. 3) and their selective binding with fAβ fragments (Fig. 2) would lead to the formation of the fAβ-T_12_P_5_-CD68 complex. Therefore, the fAβ-T_12_P_5_-CD68 complex may facilitate the transfer of fAβ into lysosomes and accelerate their clearance (Fig. 8c). Also, the interactions of fAβ with T_12_P_5_ may result in the formation of an fAβ-T_12_P_5_ complex that can be easily recognized by lysosomes as evidenced by the association of the lysosomal intracellular vesicle LAMP-1 and the fluorescently labeled T_12_P_5_(PS) (Fig. 8d). This role of T_12_P_5_ was further supported by the finding that autophagosomes were highly activated in the T_12_P_5_-co-incubated BV2 cells after acute exposure to fAβ (Figs. 8e–g, 9). In AD patients and animal models, impaired autophagosome biogenesis has been reported [111–113]. In fact, stimulation of autophagy is a therapeutic strategy that has been explored to facilitate fAβ clearance in AD animal models. In Tg2576 mice, modulation of the signaling pathways that regulate autophagosome biogenesis through genetic and pharmacological treatments was shown to alleviate memory deficits, reduce Aβ deposits, and ameliorate tau pathology [114, 115]. Interestingly, stimulating autophagy by targeting signaling pathways in a mouse model of human tauopathy was found effective to reduce neurodegeneration [114]. However, such signaling pathways are involved in other critical cellular functions including cell growth and gene translation, making this approach less effective. Gene therapies activating the expression of autophagic components effectively alleviate Aβ burden and prevent cognitive decline in AD mouse models [116, 117]. To date, there is an unmet need for a novel, translational, more specific autophagy inducer to reduce neurodegeneration without impacting cellular functions. Few reports have highlighted the role of SRs in Aβ phagocytic clearance [35, 118, 119]. However, one study suggested that SRA1 specifically participates in fAβ clearance but does not play a rate-limiting role in fAβ clearance [120]. fAβ binding via immune receptors has been shown to be insufficient to trigger degradation of internalized fAβ in primary microglia [121], while microglia in brain slices could efficiently digest some plaques [122]. Consistent with this notion, an immunotherapy has shown efficiency in reducing the fAβ deposits in APP transgenic mice lacking the key immune receptor Fc gamma receptor (FcRγ) [123]. These findings support the involvement of other receptors and mechanisms in the digestion of intracellular fAβ deposits.

To date, passive immunotherapeutic approaches, including several anti-Aβ antibodies, have been investigated for their ability to target numerous forms of Aβ [85, 124, 125]. Despite the reported success in targeting toxic Aβ plaques and improving cognitive functions, most of these therapies were abandoned during clinical trials due to the lack of efficacy or severe side effects [126]. Investigations of therapeutic agents in advanced stages of AD and their permeability across the blood–brain barrier (BBB) are the major challenges associated with AD. The capability of our NPs to cross BBB and further diffuse through the brain extracellular space will be explored in future. Parameters that facilitate NPs permeability across BBB such as endothelial cells receptor specificity, charge, and size will be considered in NPs design. Further research is needed to explore the role of multi-faceted NPs with certain surface properties to divert the clearance of fAβ through precise targeting of SRs and modulating microglial phenotype.

## Conclusions

Nanoscale therapeutics can be designed to target multiple phenomena related to microglia in conditions of AD and amyloidosis. These targets include the toxic extracellular deposition of Aβ, microglial uptake of fAβ, fAβ-mediated microglial activation, and pro-inflammatory response. Also, the upstream regulatory activation of NF-κB, and the subsequent fAβ-mediated neurotoxicity and lysosomal degradation can be modulated. Collectively, our approach holds promise for the development of multimodal therapeutics that could be leveraged to target AD progression.

### Supplementary Information

**Additional file 1**. **Figure S1**. AM-NPs accelerate lysosomal clearance of fAβ.

## Data Availability

All data analyzed during the current study are available from the corresponding authors on reasonable request. Also, research materials will also be made available when they are required.

## Abbreviations

AD: Alzheimer's disease
fAβ: Fibril amyloid beta
AMs: Amphiphilic macromolecules
SRs: Scavenger receptors
CNS: Central nervous system
SRA-1: Scavenger receptors class A-1
T_12_ P_5_: Tartaric acid-derived shells
M_12_P_5_: Mucic acid-derived shells
PEG: Polyethylene glycol
FNP: Flash nanoprecipitation
Th-T: Thioflavin-T
TEM: Transmission electron microscopy
iNOS: Inducible nitric oxide synthase
TNF-α: Tumor necrosis factor alpha
NO: Nitric oxide
LAMP-1: Lysosomal-associated protein
