# Supplementary material for: Nanotechnology for microglial targeting and inhibition of neuroinflammation underlying Alzheimer’s pathology
Source: Transl Neurodegener. 2024 Jan 4;13:2. doi: 10.1186/s40035-023-00393-7 (PMC10765804; doi:10.1186/s40035-023-00393-7)
Supplement: Supplementary file 1 — Additional file 1. Figure S1. AM-NPs accelerate lysosomal clearance of fAβ. [file 40035_2023_393_MOESM1_ESM.docx]

Supplemental Information

**Nanotechnology for Microglial Targeting and Inhibition of Neuroinflammation for Alzheimer’s Pathology**

*Hoda M. Gebril ^a^***, Aravind Aryasomayajula ^a^,* [*Mariana Reis Nogueira de Lima*](https://acs.digitellinc.com/speakers/view/328029) *^c^, Kathryn E. Uhrich ^c^, Prabhas V. Moghe ^a,b^**

^a^ Department of Biomedical Engineering, 599 Taylor Rd., Rutgers University, NJ 08854, USA

^b^ Department of Chemical and Biochemical Engineering, 98 Brett Rd., Rutgers University, NJ 08854, USA

^c^ Department of Chemistry, 501 Big Springs Rd., University of California, Riverside, CA 92507, USA

** Corresponding authors:*

*Hoda M. Gebril:* [*hoda.gebril@rutgers.edu*](mailto:hoda.gebril@rutgers.edu)

*Prabhas V. Moghe:* [*moghe@rutgers.edu*](mailto:moghe@rutgers.edu)


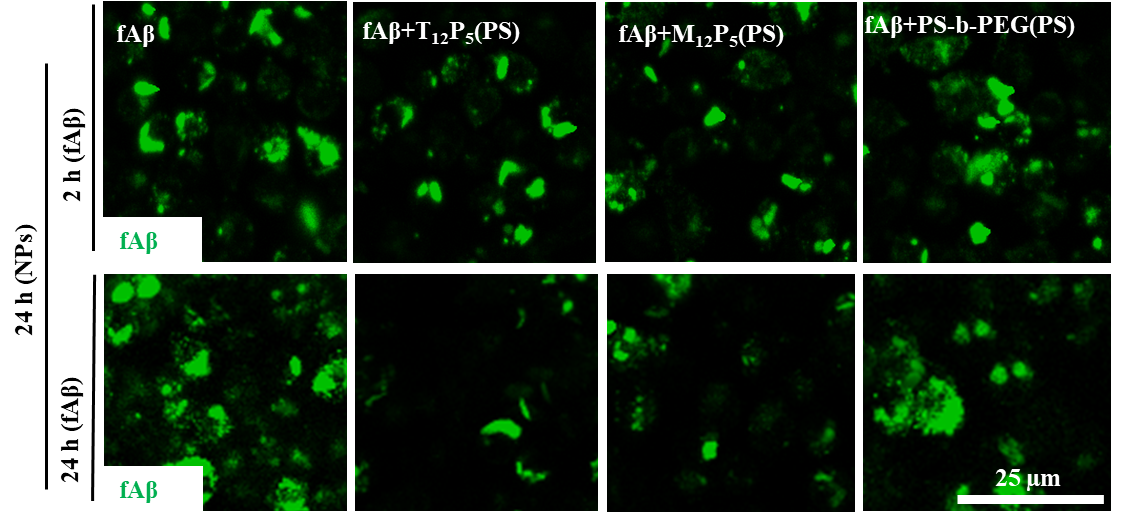


**Figure S1. AM-NPs accelerate lysosomal clearance of fAβ.** BV2 microglia incubated with NPs for 24 h then co-incubated with Alexa Fluor 488-labeled fAβ for 2 h or 24 h. Lysosomes in treated live BV2 microglia were stained with 70 µM Lysotracker for 30 min. Cells were then fixed using 4% PFA. Representative confocal microscopy images showing fAβ-positive (green) at 2 h (top row) and 24 h (bottom row) after fAβ treatment.
